# An EGFR-Targeted Fusogenic Tandem Peptide for siRNA Delivery in Glioblastoma

**DOI:** 10.3390/pharmaceutics18091086

**Published:** 2026-08-28

**Authors:** Jessica R. Boulos, Karen E. Russi, Jordan Kinnitt, Daphne Gomez Escudero, Tyler Willis, Jorrian Abadeer, Emalee Mann, Aaron Cristina Anderson, Angela Alexander-Bryant

**Affiliations:** Department of Bioengineering, Nanobiotechnology Laboratory, Clemson University, Clemson, SC 29631, USA; jboulos@clemson.edu (J.R.B.);

**Keywords:** peptide-based delivery system, RNA interference, targeted gene therapy, glioblastoma, siRNA

## Abstract

**Background/Objectives**: RNA interference (RNAi) represents a promising therapeutic approach for silencing oncogenes involved in cancer progression by utilizing small interfering RNA (siRNA). However, siRNA requires an efficient delivery system to overcome cellular uptake and endosomal escape barriers. This study aimed to evaluate a multifunctional tandem peptide, GE11-599, designed to enhance the targeted delivery of siRNA and maintain its bioactivity in glioblastoma (GBM) cells. **Methods**: The GE11-599 peptide, consisting of an EGFR-targeting GE11 motif and a 599 fusogenic domain, was complexed with siRNA via electrostatic interactions to form nanoparticles. We assessed nanoparticle physicochemical properties, protection of siRNA from serum and RNase degradation, and cellular uptake in two GBM cell lines (U118MG and U87MG). Mechanistic studies evaluated receptor-mediated endocytosis and the subsequent escape from endosomes. Functional assays quantified *STAT3* gene silencing and downstream effects on cell migration following treatment with GE11-599–siSTAT3 complexes. **Results**: GE11-599 formed positively charged, monodisperse nanoparticles capable of protecting siRNA from degradation. The tandem peptide significantly enhanced cellular internalization through EGFR-mediated endocytosis and facilitated endosomal escape of siRNA. Treatment with GE11-599–siSTAT3 resulted in robust gene silencing, achieving up to an 80% reduction in *STAT3* mRNA expression. Downstream functional assessment showed a 40% decrease in migration in GBM cells treated with GE11-599–siSTAT3 complexes. **Conclusions**: The GE11-599 tandem peptide effectively enhances cell-specific internalization and endosomal escape of siRNA in GBM cells, resulting in increased siRNA bioactivity and functional gene silencing. These findings support GE11-599 as a promising siRNA delivery platform for targeting EGFR-expressing cancers.

## 1. Introduction

Glioblastoma (GBM) is the most common malignant brain cancer, making up 47% of central nervous system cancers [1]. Treatment for GBM typically involves surgical resection, chemotherapy, and radiation; however, these treatments result in low survival and can harm healthy tissue [2]. Even with aggressive treatment, patients with GBM have a median survival rate of 8 months and a five-year survival rate of 6.9% [3,4]. Thus, new treatment strategies are needed to improve patients’ prognosis. A promising avenue for advancing the treatment of many cancers, including GBM, is RNA interference (RNAi), which can target oncogenes that promote cancer progression [5]. This technique utilizes short hairpin RNA or small interfering RNA (siRNA) to target messenger RNA (mRNA) in a sequence-specific manner, inhibiting protein translation and silencing overexpressed oncogenes [6]. Although RNAi has therapeutic potential, its clinical translation faces challenges due to siRNA delivery barriers, including low cellular uptake, non-specific internalization, and endosomal entrapment [7]. To overcome these barriers, effective delivery systems are required for sufficient internalization of bioactive siRNA.

Peptides have been widely investigated as delivery platforms due to their affordability, biocompatibility, and modular tunability for diverse biomedical applications [8]. Their functional versatility enables the rational incorporation of targeting, cell-penetrating, and stimuli-responsive motifs to address multiple biological barriers to delivery [8]. Targeting peptides promote selective cellular uptake by engaging specific cell-surface receptors through sequence-defined interactions. Ligand–receptor binding triggers receptor-mediated endocytosis, facilitating internalization of the cargo [9]. Cell-penetrating peptides are typically cationic and increase internalization through electrostatic interactions between the peptide and negatively charged cell membrane [10]. Lastly, stimuli-responsive sequences, such as fusogenic peptides, are activated by environmental triggers to facilitate endosomal escape, enabling cytoplasmic release and biological activity of their cargo [11]. Following endocytosis, acidification of the endosomal compartment induces a conformational change in the fusogenic peptide, typically adopting an alpha-helical secondary structure that drives insertion into the endosomal membrane and destabilization, resulting in endosomal escape [12]. Although each of these peptides has been studied individually, there has been limited focus on evaluating the combined effects of these peptides in a delivery system. Most research on multifunctional tandem peptides focuses on the combination of targeting and cell-penetrating peptides, which enhances cell-specific internalization. However, endosomal entrapment remains a significant barrier, resulting in reduced efficacy of the internalized cargo [13].

To address this barrier, we hypothesized that a tandem peptide combining a targeting peptide with a fusogenic peptide sequence could enhance siRNA delivery and bioactivity for the treatment of GBM. We developed a tandem peptide comprised of an epidermal growth factor receptor (EGFR) targeting sequence and a known fusogenic peptide. The targeting sequence is the GE11 peptide, specifically targeting EGFR, which is upregulated in GBM and plays a role in cell growth, differentiation, and migration [14]. Previous research has shown that the GE11 peptide can bind EGFR with high specificity without activating the receptor, preventing a mitogenic effect [15]. GE11 has been conjugated to other delivery systems, such as liposomes or polymersomes, for receptor-specific internalization across various cell types, including ovarian and lung cancer [16,17,18]. The fusogenic sequence consists of the 599 peptide, derived from the INF-7 virus, which undergoes a pH-dependent conformational change in the endosome to release bioactive siRNA into the cytoplasm [19]. The inclusion of a nona(D-arginine) tail allows self-assembly through electrostatic complexation with siRNA. The 599 peptide has been demonstrated to be an efficient siRNA delivery vehicle, inducing endosomal escape and permitting gene silencing of CIP2A in oral cancer cells in vitro and in vivo [20]. We also included the nona(D-arginine) tail on the C-terminus of the GE11 targeting sequence (GE11R9) and the GE11-599 tandem peptide to create a positive charge for electrostatic complexation with negatively charged siRNA [21]. The GE11R9 and 599 peptides are evaluated individually as controls to compare the functionality of the tandem GE11-599 peptide. Our gene target of interest is Signal Transducer and Activator of Transcription 3 (*STAT3*), which regulates cell proliferation, migration, apoptosis, differentiation, and angiogenesis [22]. Approximately 90% of GBM tissues and cells exhibit *STAT3* activation, making it an ideal target for gene therapy [23].

We demonstrated that the GE11-599 multifunctional tandem peptide retains the functionality of its individual sequences, acting as an efficient delivery carrier for bioactive siRNA. The peptide electrostatically complexes siRNA to form positively charged monodisperse nanocomplexes and protects siRNA from enzymatic degradation. The GE11-599 peptide successfully internalized siRNA into two GBM cell lines through EGFR receptor-mediated endocytosis. Furthermore, by facilitating endosomal escape of bioactive siRNA, the tandem peptide significantly silenced the *STAT3* gene, reducing GBM cellular migration up to 40%. Additionally, we found that the GE11-599 tandem peptide enabled increased efficacy of bioactive siRNA cargo compared to the targeting and fusogenic sequences alone by allowing cell-specific internalization and efficient endosomal escape within a single sequence. Overall, this study demonstrated the feasibility of multifunctional tandem peptides in overcoming several barriers associated with siRNA delivery.

## 2. Materials and Methods

### 2.1. Cell Culture

Human glioblastoma cell lines U118MG and U87MG were obtained from American Type Culture Collection (ATCC, Manassas, VA, USA) and cultured in Dulbecco’s Modified Eagle Medium and Eagle’s Minimum Essential Medium, respectively, supplemented with 10% fetal bovine serum and 1% penicillin/streptomycin antibiotic solution (Corning, Corning, NY, USA). Healthy cell lines, HEK-293 human embryonic kidney cells and HMC3 human microglial cells, were obtained from ATCC and cultured in Dulbecco’s Modified Eagle Medium and Eagle’s Minimum Essential Medium, respectively, supplemented with 10% fetal bovine serum and 1% penicillin/streptomycin antibiotic solution (Corning). All cultures were incubated at 37 °C and 5% CO_2_.

### 2.2. Peptide Synthesis

The GE11R9 (YHWYGYTPQNVIGGGG-(d)RRRRRRRRR-K), 599 (GLFEAIEGFIENGWEGMIDGWYGGGG-(d)RRRRRRRRR-K), and GE11-599 (YHWYGYTPQNVIGGGGGLFEAIEGFIENGWEGMIDGWYGGGG-(d)RRRRRRRRR-K) peptides were synthesized, purified (95% purity), and analyzed via high-performance liquid chromatography by Lifetein (Lifetein LLC, Somerset, NJ, USA). Lyophilized peptides were resuspended in ultrapure H_2_O to a 10 mg/mL stock solution. Working solutions were prepared at 1 mg/mL by diluting the stock peptide (1:10) with RNase-free water. All peptide solutions were stored at −80 °C.

### 2.3. Gel Electrophoresis/Protection

All peptides were complexed with 10 mM siGENOME non-targeting siRNA#5 (siNT#5, GE Healthcare Dharmacon, Lafayette, CO, USA) through electrostatic complexation in RNase-free water for 20 min at room temperature. Peptide complexes were made at varying amine:phosphate (N:P) ratios to determine the minimal amount of peptide needed for complete complexation with siRNA. Complexes were loaded into a 2% agarose gel, electrophoresed at 100 V for 35 min, stained with ethidium bromide, and imaged using the ChemiDoc Imaging System (Bio-Rad Laboratories, Hercules, CA, USA). To determine the ability of the peptides to protect siRNA from degradation, complexes were formed as previously described and incubated in RNase-free water or treated with either 50% *v*/*v* FBS or 0.15 mg/mL of RNase A for 1 h at 37 °C. Following incubation, complexes were dissociated with a 6% sodium dodecyl sulfate (SDS) solution and electrophoresed as described.

### 2.4. Dynamic Light Scattering

Peptide-siNT#5 complexes were formed at 0.2 mg/mL of peptide in RNase-free water as described at 80:1 N:P ratios. The zeta potential and hydrodynamic size of the complexes were determined using a Zetasizer Nano ZS (Malvern Panalytical, Malvern, UK), and the data were analyzed using Malvern’s Zetasizer software v8.02.

### 2.5. Viability Studies

All cell lines were seeded at 10,000 cells/well in a 96-well plate and allowed to attach overnight. For cytocompatibility assays, peptides were complexed with siNT#5 at 20:1–80:1 N:P ratios and incubated with U118MG, U87MG, HEK293, and HMC3 cell lines for 48 h in base media supplemented with 10% FBS (10% FBS transfection media). Viability was measured through an MTS Assay using CellTiter 96 Aqueous One Solution Cell Proliferation Assay (Promega, Madison, WI, USA). The reagent was added to the cells for 3 h according to the manufacturer’s protocol, and absorbance was read using a BioTek Synergy Gen5 plate reader (BioTek, Winooski, VT, USA) at 490 nm. For the downstream viability assay, GE11-599 peptide was complexed with either siNT#5 or siSTAT3 at a 60:1 and 80:1 ratio. Complexes were incubated with U11MG and U87MG cell lines for 48 h in 10% transfection media, and viability was assessed as previously described.

### 2.6. Fluorescent Microscopy

Cells were seeded at 100,000 cells/well in a 12-well plate and allowed to attach overnight. Peptides were complexed with Cy5-labeled siNT#5 (Dharmacon) at 20:1–80:1 N:P ratios and incubated with U118MG and U87MG cell lines in 10% FBS transfection media for 4 h. Following incubation, the media was aspirated, and cells were washed with 1× PBS and stained with Hoechst 3342 (Thermo Fisher Scientific, Waltham, MA, USA) for 20 min. Imaging was conducted using the EVOS FL microscope (Thermo Fisher Scientific) at 20× magnification.

### 2.7. Flow Cytometry

Cells were seeded at 50,000 cells/well in a 24-well plate and allowed to attach overnight. Peptides were complexed with Cy5-siNT#5 at 20:1–80:1 N:P ratios and incubated with U118MG and U87MG cell lines in 10% transfection media for 4 h. Following incubation, cells were washed with 1× PBS, detached using 0.25% Trypsin to remove loosely and receptor-bound complexes, and resuspended in 1× PBS for analysis via the Attune NXT acoustic flow cytometer (Thermo Fisher Scientific) with the BL1 laser. Data analysis was conducted through FlowJo software v2.7 (BD Biosciences, Franklin Lakes, NJ, USA).

### 2.8. Fluorophore Quenching Assay

GE11-599 peptide was complexed with Dy547 siRNA at N:P ratios of 20:1, 40:1, 60:1, and 80:1 as previously described. Complexes were prepared and transferred to a 96-well black-walled plate. Fluorescence intensity was measured using a BioTek Synergy Gen5 plate reader equipped with a red fluorescence filter cube (586/15 nm excitation and 647/57 emission wavelengths).

### 2.9. EGFR Competitive Binding Assay

Cells were seeded at 10,000 cells/well in a 96-well plate and allowed to attach overnight. The epidermal growth factor receptor (EGFR) was blocked using an anti-EGFR antibody (Cat No. MAB9577, Bio-Techne, Minneapolis, MN, USA), a Cetuximab bioequivalent, at 20 mg/mL in 1× PBS for 1 h at 37 °C. Following blocking, cells were treated with Cy5-siNT#5 complexed with GE11R9, 599, or GE11-599 peptide at an 80:1 N:P ratio in 10% transfection media for 3 h. Following treatment, the media was aspirated, the cells were washed with 1× PBS, and Hoescht 33342 stain was added for 20 min. Imaging was conducted with the EVOS FL microscope at 20× magnification.

### 2.10. Immunofluorescent Staining

Cells were seeded at 10,000 cells/well in a 96-well plate and allowed to attach overnight. Peptides were complexed with Cy5-siNT#5 at N:P ratios of 20:1–80:1 and incubated with U118MG and U87MG cell lines in 10% FBS transfection media for 4 h. Following incubation, cells were fixed with 4% paraformaldehyde in 1× PBS for 15 min and permeabilized with 0.5% Triton X-100 in 1× PBS for 15 min at room temperature. Cells were washed three times with 1× PBS, and blocking was performed with 0.5% BSA in 1× PBS for 30 min at room temperature. Primary antibody for Early Endosome Antigen 1 (EEA1, Thermo Fisher Scientific) was added at a 1:100 dilution with 1× PBS for 1 h at 4 °C in the dark. Following incubation with the primary antibody, cells were washed three times with 1× PBS. Alexa-488 anti-rabbit secondary antibody (Thermo Fisher Scientific) was added for 1 h at room temperature in the dark. Following three washes with 1× PBS, Hoescht 33342 was added to the cells to stain the nuclei, and imaging was conducted with the EVOS FL microscope at 20× magnification.

Colocalization between EEA1-positive early endosomes and Cy5-labeled siRNA was quantified using threshold Manders’s colocalization coefficients, calculated in Fiji using the Coloc2 plugin, to assess endosomal escape across N:P ratios and peptide formulations.

### 2.11. qPCR

Cells were seeded at 50,000 cells/well in a 24-well plate and allowed to attach overnight. Peptides were complexed with siNT#5 or STAT3 siRNA (#sc-29493, Santa Cruz Biotechnology, Dallas, TX, USA) at 20:1-80:1 N:P ratios and incubated in 10% FBS transfection media for 48 h. Following treatment, RNA isolation was performed using the Qiagen Mini-RNeasy Kit (Qiagen, Hilden, Germany) according to the manufacturer’s protocol. RNA quantification and purity were analyzed using the Gen5 protocol on a Take3 plate (BioTek) using the plate reader. RNA was reverse-transcribed into cDNA for qPCR analysis using the High-Capacity cDNA Reverse Transcription Kit (Applied Biosystems, Waltham, MA, USA) according to the manufacturer’s protocol. Quantitative real-time PCR was performed using a QuantStudio 3 qPCR system (Applied Biosystems) with Applied Biosystems TaqMan FAST Master Mix and Thermo Fisher probes: 18s (ID# Hs99999901_s1) and STAT3 (ID# Hs00374280_m1).

### 2.12. Scratch Migration Assay

U118MG and U87MG cells were seeded at 75,000 cells/well in a 24-well plate and allowed to attach overnight. GE11-599 peptide was complexed with siNT#5 or siSTAT3 at 60:1 and 80:1 ratios and incubated with both cell lines for 48 h. Following treatment, a horizontal scratch was made in the cell monolayer using a 1000 μL pipette tip, and the area was gently washed with 1× PBS to remove any floating cells. Cells were imaged with an EVOS FL microscope using 4× magnification immediately after creating the scratch and at 24-h and 48-h post-scratch. Measurements across the horizontal axis were taken for each image, and the wound closure percentage was calculated at each time point using the equation below, where $t_{i}$ is the distance at a given time point and $t_{0}$ is the distance at the initial time point, as determined using ImageJ analysis software v 1.54t [24].

$$\%\;Wound\;Healing=\frac{t_{i}-t_{0}}{t_{i}}\times 100$$

### 2.13. Statistical Analysis

Statistical analyses were performed through GraphPad Prism version 10.0.0. Data are presented as mean ± standard error of the mean (SEM) of three independent experiments. Comparison between different N:P ratios of a single peptide and untreated cells was analyzed using one-way ANOVA followed by Dunnett’s post hoc test. Comparisons involving N:P ratios across multiple peptides against untreated cells were performed using two-way ANOVA with post hoc Dunnett’s comparisons. A two-way ANOVA with Tukey’s multiple comparisons was applied for comparisons across all treatment groups. Significance was indicated where *p* ≤ 0.05.

## 3. Results

### 3.1. The GE11R9, 599, and GE11-599 Peptides Complex with siRNA and Protect siRNA from Degradation

**Figure 1:**
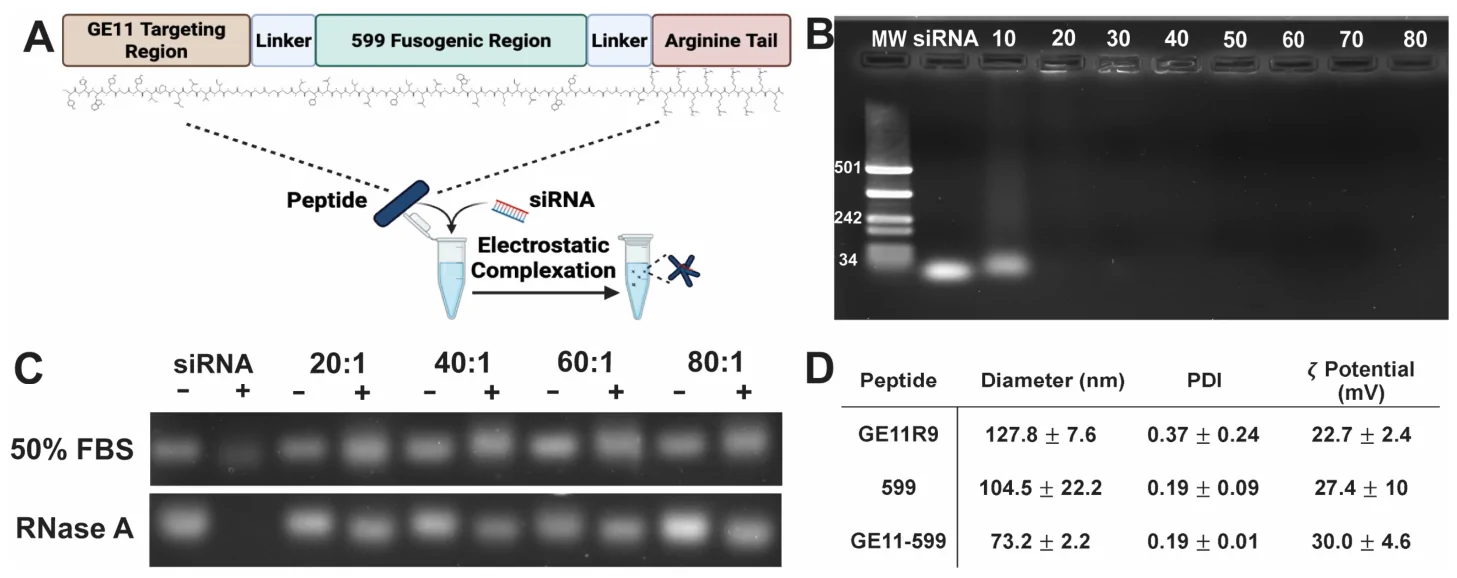
**GE11-599 Peptide Characterization.** (**A**) GE11-599 peptide design and electrostatic complexation with siRNA. (**B**) Gel electrophoresis of GE11-599 at increasing N:P ratios with intact, free siRNA indicated by white banding. (**C**) Protection assays of GE11-599 peptide–siRNA complexes incubated with (+) 50% FBS or 0.15 mg/mL RNase A or (−) RNase-free H_2_O. (**D**) Dynamic light scattering analysis of diameter, polydispersity index (PDI), and zeta potential for each peptide.

The GE11R9, 599, and GE11-599 peptides were designed with a cationic nona-d-arginine tail to allow electrostatic complexation with negatively charged siRNAs (Figures S1A and S2A and Figure 1A). Peptide/siRNA complexes were formed at varying N:P ratios, and agarose gel electrophoresis was used to determine the minimum ratio necessary for complete complexation for each peptide, where white banding indicates free siRNA. For the GE11R9 peptide, partial complexation with siNT#5 occurred at N:P ratios of 10:1–30:1, as noted by the partial lack of banding in these wells. However, at an N:P ratio of 40:1, complete complexation occurred (Figure S1B). The 599 and GE11-599 peptides complexed with siRNA at N:P ratios of 10:1 and 20:1, respectively (Figure S2B and Figure 1B). Therefore, as 20:1 represented the minimum N:P complexation ratio for our GE11-599 peptide, ratios of 20:1–80:1 were selected as a comprehensive range for further experiments with all peptides. To determine the capability of the peptides to protect siRNA from degradation, complexes were incubated in either RNase-free water (−) or a degradative environment (+): 50% FBS to mimic physiological conditions and RNase A to represent harsher enzymatic degradation. All peptides successfully protected siRNA from degradation in both environments at N:P ratios ranging from 20:1–80:1, as evidenced by the intact siRNA banding following incubation (Figure 1C and Figures S1C and S2C), with 599 and GE11-599 peptides providing superior protection compared to GE11R9.

Following confirmation of complexation, dynamic light scattering analysis was conducted on the peptide–siRNA complexes to analyze the nanoparticle size, distribution, and surface charge. For each peptide, complexes with siNT#5 were prepared at an 80:1 ratio, corresponding to the maximum ratio assessed in this study. The GE11R9 nanocomplexes exhibited an average hydrodynamic diameter of 127.8 ± 7.6 nm with a zeta potential of 22.7 mV. The 599 peptide complexes formed the largest and most positively charged nanoparticles, with a diameter of 104.5 ± 22.2 nm and a zeta potential of 27.4 mV. Finally, the GE11-599 peptide formed nanoparticles with a diameter of 73.2 ± 2.2 nm and a zeta potential of 30 mV. Additionally, the data indicate that all three peptides form relatively monodisperse nanocomplexes with siRNA, as evidenced by polydispersity index (PDI) values of 0.37 or lower for each peptide–siRNA formulation (Figure 1D). Representative size distribution profiles for all formulations at the 80:1 N:P ratio are provided in Figure S3.

### 3.2. All Peptides Are Cytocompatible in Glioblastoma and Healthy Cell Lines

**Figure 2:**
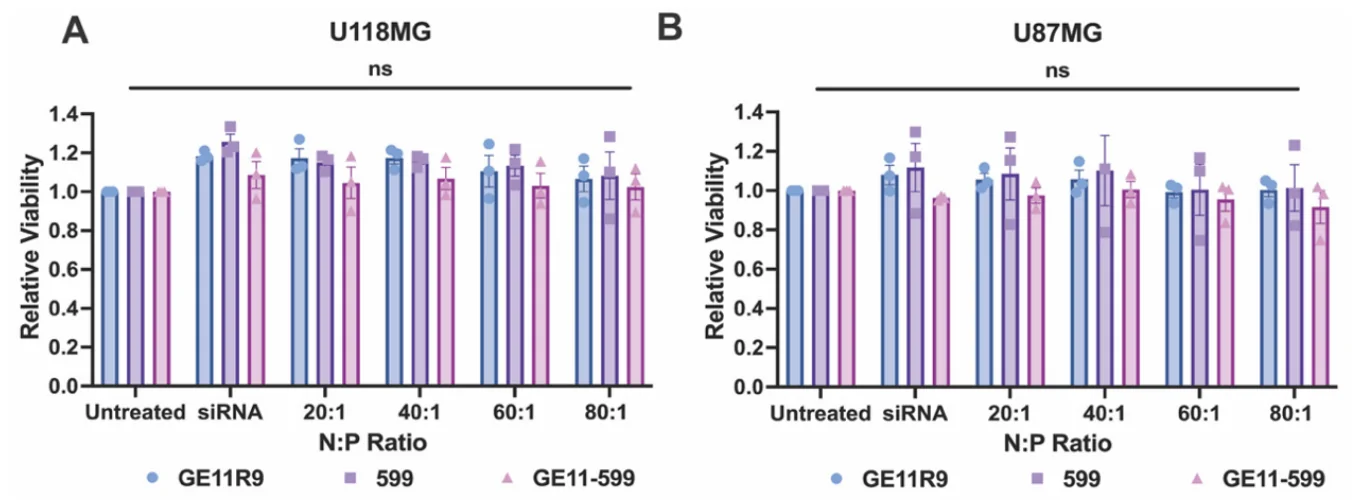
**Peptide Cytocompatibility in GBM cells.** Cellular viability after 48-h treatment with peptide–siNT#5 complexes at varying N:P ratios in (**A**) U118MG and (**B**) U87MG cells. Data are mean ± SEM of three independent experiments performed in triplicate, where ns = not significant compared to untreated cells analyzed via two-way ANOVA using Dunnett’s multiple comparisons test.

To assess the cytocompatibility of the GE11R9, 599, and GE11-599 delivery vehicles, complexes were prepared with each peptide and siNT#5 at N:P ratios of 20:1–80:1 and incubated with glioblastoma cell lines. In U118MG and U87MG cells, all peptide complexes induced minimal changes in viability compared to untreated cells, indicating that the peptides are cytocompatible after a 48-h transfection (Figure 2A,B). To further investigate the cytocompatibility of the GE11-599 peptide in healthy cell lines, HEK-293 and HMC3 cells were treated with peptide/siRNA complexes for 48 h using the same N:P ratios. Similar to the GBM cell lines, treatment with GE11-599 peptide complexes had minimal effect on the viability of the cells, indicating cytocompatibility in healthy cell lines (Figure S4).

### 3.3. All Peptides Internalize siRNA into Glioblastoma Cell Lines

**Figure 3:**
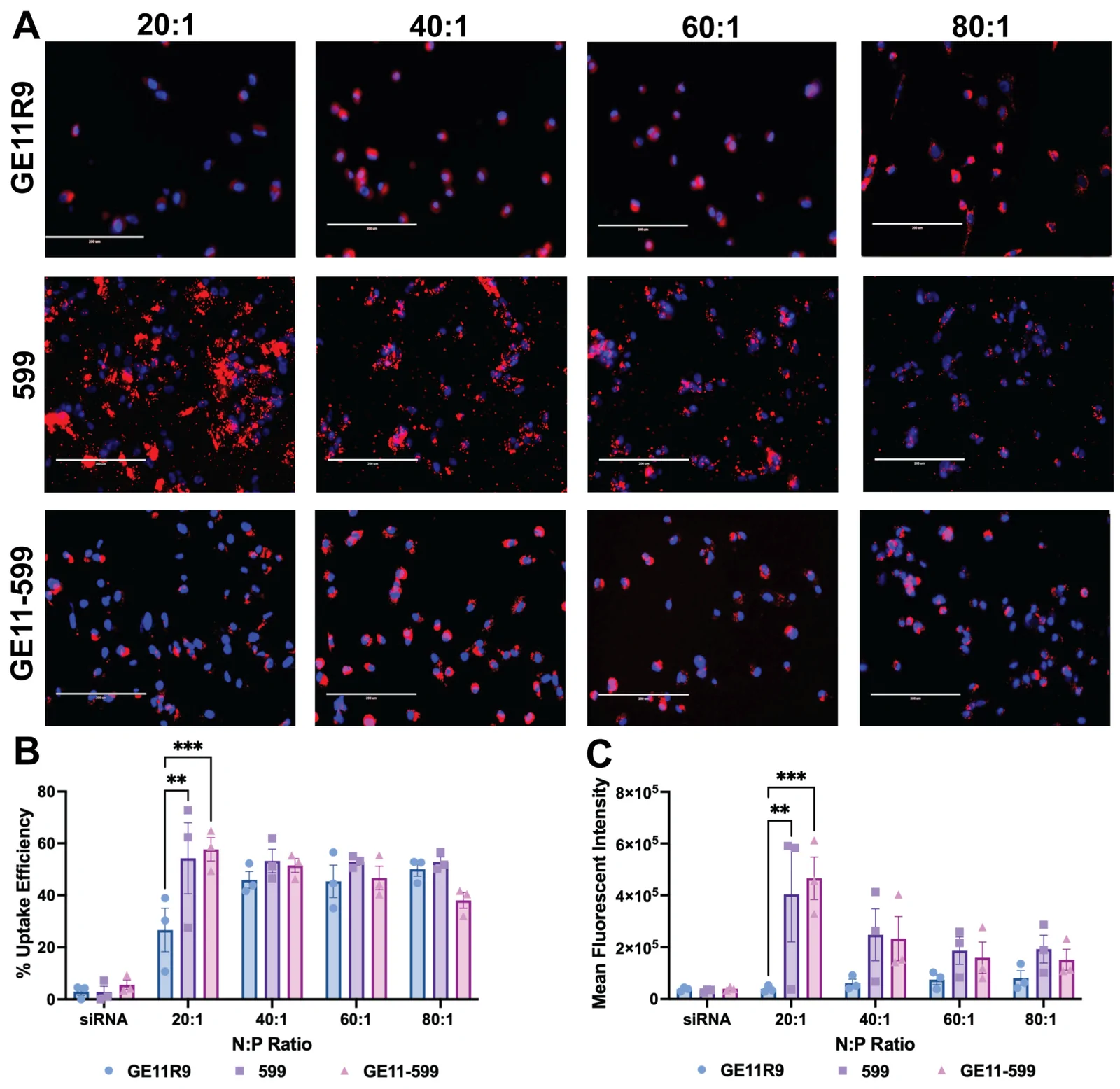
**Peptide–siRNA Cellular Internalization in U118MG GBM Cells.** (**A**) Fluorescent microscopy imaging of siRNA internalization in U118MG cells treated with peptide–Cy5-siNT#5 (red) complexes for 4 h. Cell nuclei stained with Hoechst 33342 (blue). Scale bar = 200 μm. (**B**) Flow cytometric quantification of peptide-mediated siRNA internalization after 4 h. (**C**) Mean fluorescent intensity of siRNA internalized by peptide complexes after 4 h. Data are mean ± SEM of three independent experiments analyzed via two-way ANOVA with Tukey’s multiple comparisons test across peptides within each ratio, where ** *p* ≤ 0.01 and *** *p* ≤ 0.001.

Fluorescent microscopy imaging and flow cytometry were utilized to ensure the peptides could facilitate the internalization of siRNA into GBM cells. GE11R9, 599, and GE11-599 peptides were complexed with Cy5-siNT#5 at N:P ratios of 20:1–80:1 for incubation with GMB cells. In the U118MG cells, fluorescence imaging confirmed the internalization of siRNA at all ratios of each peptide (Figure 3A). Flow cytometry revealed that at a 20:1 ratio, GE11R9 complexes exhibited 26% internalization compared to 54% for 599 and 58% for GE11-599 complexes. At N:P ratios of 40:1 to 80:1, the peptides facilitated similar levels of siRNA internalization, with approximately 45–55% uptake (Figure 3B). The flow gating strategy for U118MG cells is provided in Figure S5A. Mean fluorescent intensity (MFI), which reflects the amount of internalized siRNA, was similar for GE11R9 complexes and siRNA alone at an N:P ratio of 20:1, and increased 1.6–2.1-fold at ratios of 40–80:1 (Figure 3C). The 599 and GE11-599 peptides displayed similar trends, both exhibiting the highest MFI at a 20:1 N:P ratio, corresponding to 11- and 12.6-fold increases, respectively, compared to siRNA alone. As the ratio increased, the MFI decreased for both peptides, reaching approximately 6.5-fold at a 40:1 ratio. At 60:1 and 80:1 N:P ratios, MFI further declined to 4.7-fold relative to free siRNA for both 599 and GE11-599.

**Figure 4:**
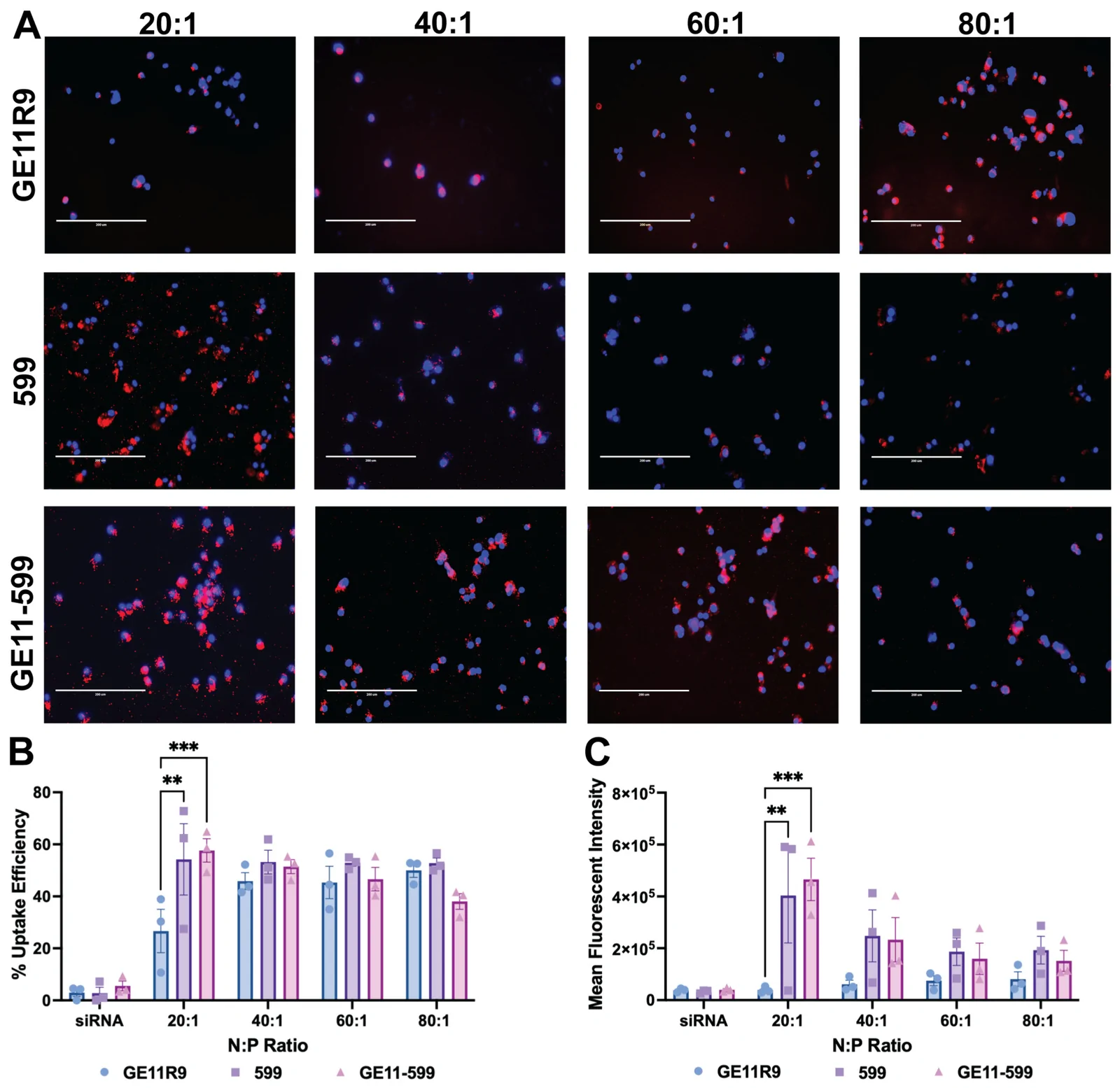
**Peptide–siRNA Cellular Internalization in U87MG GBM Cells.** (**A**) Fluorescent microscopy imaging of siRNA internalization in U87MG cells treated with peptide–Cy5-siNT#5 (red) complexes for 4 h. Cell nuclei stained with Hoechst 33342 (blue). Scale bar = 200 μm. (**B**) Flow cytometric quantification of peptide-mediated siRNA internalization after 4 h. (**C**) Mean fluorescent intensity of siRNA internalized by peptide complexes after 4 h. Data are mean ± SEM of three independent experiments analyzed via two-way ANOVA with Tukey’s multiple comparisons test across peptides within each ratio, where ** *p* ≤ 0.01 and *** *p* ≤ 0.001.

Uptake analysis of another GBM cell line, U87MG, further confirmed the ability of the peptides to facilitate the internalization of siRNA (Figure 4A). Flow cytometry results showed that at a 20:1 N:P ratio, GE11R9 complexes exhibited 38% internalization compared to 57% for 599 and GE11-599. At 40:1–80:1 N:P ratios, the peptides facilitated similar levels of siRNA internalization, with approximately 45–55% uptake (Figure 4B). The flow gating strategy for U87MG cells is provided in Figure S5B. Relative to siRNA alone, GE11R9-mediated delivery resulted in modest increases in MFI, corresponding to 2.5-fold at 20:1, 3.5-fold at 40:1 and 60:1, and a 6.5-fold at 80:1 N:P ratios (Figure 4C). As seen in U118MG cells, the 20:1 ratio of the 599 and GE11-599 peptides exhibited the highest MFI, with a 24.1- and 39.1-fold increase, respectively. At a 40:1 N:P ratio, the 599 peptide complexes exhibited a 14-fold increase in MFI, and the GE11-599 complexes displayed a 19-fold increase in comparison to free siRNA. The 60:1 and 80:1 ratios of both peptides exhibited similar MFI, with a 9.5-fold increase compared to siRNA.

To assess whether increasing peptide density at higher N:P ratios induces self-quenching of the fluorophore conjugated to siRNA, Dy547-labeled siRNA was complexed with GE11-599 at N:P ratios of 20:1, 40:1, 60:1, and 80:1, and fluorescence intensity was measured using a BioTek Synergy Gen5 plate reader with the red fluorescence filter cube. No significant reduction in fluorescence intensity was observed as the N:P ratio increased, indicating that complexation-associated peptide packing does not induce self-quenching under the tested conditions. This suggests that fluorophore quenching does not account for the observed MFI trend across N:P ratios (Figure S6).

### 3.4. The GE11R9 and GE11-599 Peptides Specifically Target the Epidermal Growth Factor Receptor

**Figure 5:**
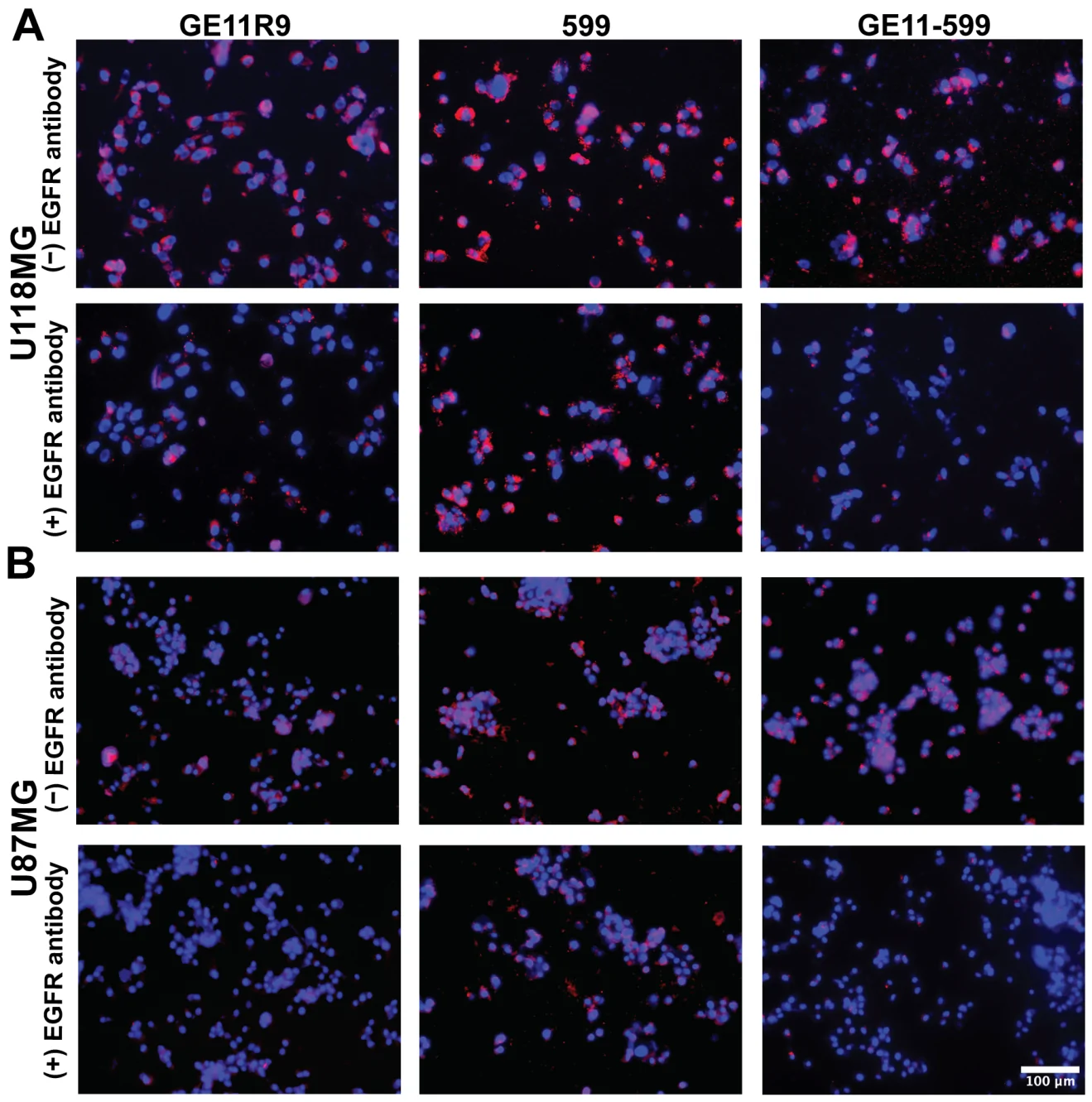
**EGFR Competitive Binding of Peptide–siRNA Complexes.** (**A**) U118MG and (**B**) U87MG cells were pretreated with either PBS (−EGFR antibody) or a Cetuximab biosimilar (+EGFR antibody) for 1 h followed by treatment with peptide–Cy5-siNT#5 (red) complexes for 3 h and imaged via fluorescent microscopy. Cell nuclei stained with Hoescht 33342 (blue). Scale bar = 100 μm.

To confirm the internalization route for our peptides via EGFR binding, we conducted a competitive binding assay by pretreating GBM cells with an antibody to block EGFR before treating the cells with peptide–siRNA complexes. GBM cells were pretreated with PBS (−EGFR antibody) or a Cetuximab biosimilar (+EGFR antibody) and then treated with peptide–Cy5-siNT#5 complexes at an 80:1 N:P ratio. Cetuximab is a monoclonal antibody used to treat various cancers by targeting EGFR and blocking the binding of ligands that activate growth pathways [25]. The biosimilar antibody contains the same variable regions that bind EGFR, efficiently blocking the receptor [26]. Fluorescent microscopy was used to compare internalization in cells treated with and without the EGFR antibody in two GBM cell lines, U118MG and U87MG (Figure 5A,B). The GE11R9 peptide has been confirmed to target EGFR; so, we included an investigation of this peptide in our GBM cells as a positive control [15]. When comparing pretreatment with and without the EGFR antibody, we observed a decrease in Cy5-siNT#5 uptake with EGFR antibody pretreatment, confirming that the GE11R9 peptide is specific for EGFR and utilizes this receptor as its internalization route. The 599 peptide, which lacks targeting capability, facilitated similar siRNA internalization regardless of anti-EGFR antibody treatment in U118MG and U87MG cells. The GE11-599 tandem peptide performed similarly to the GE11R9 peptide, where reduced siRNA internalization was observed in cells pretreated with an EGFR antibody compared to cells without the antibody, indicating specificity for EGFR as a route of internalization in both GBM cell lines. ImageJ was used to quantify siRNA fluorescence intensity. Treatment with the anti-EGFR antibody resulted in a significant reduction in siRNA uptake mediated by the GE11-599 complexes compared to untreated controls, with approximately 10-fold and 13-fold decreases observed in U118MG and U87MG cells, respectively (Figure S7).

### 3.5. The 599 and GE11-599 Peptides Facilitate the Endosomal Escape of Cargo into the Cytoplasm

**Figure 6:**
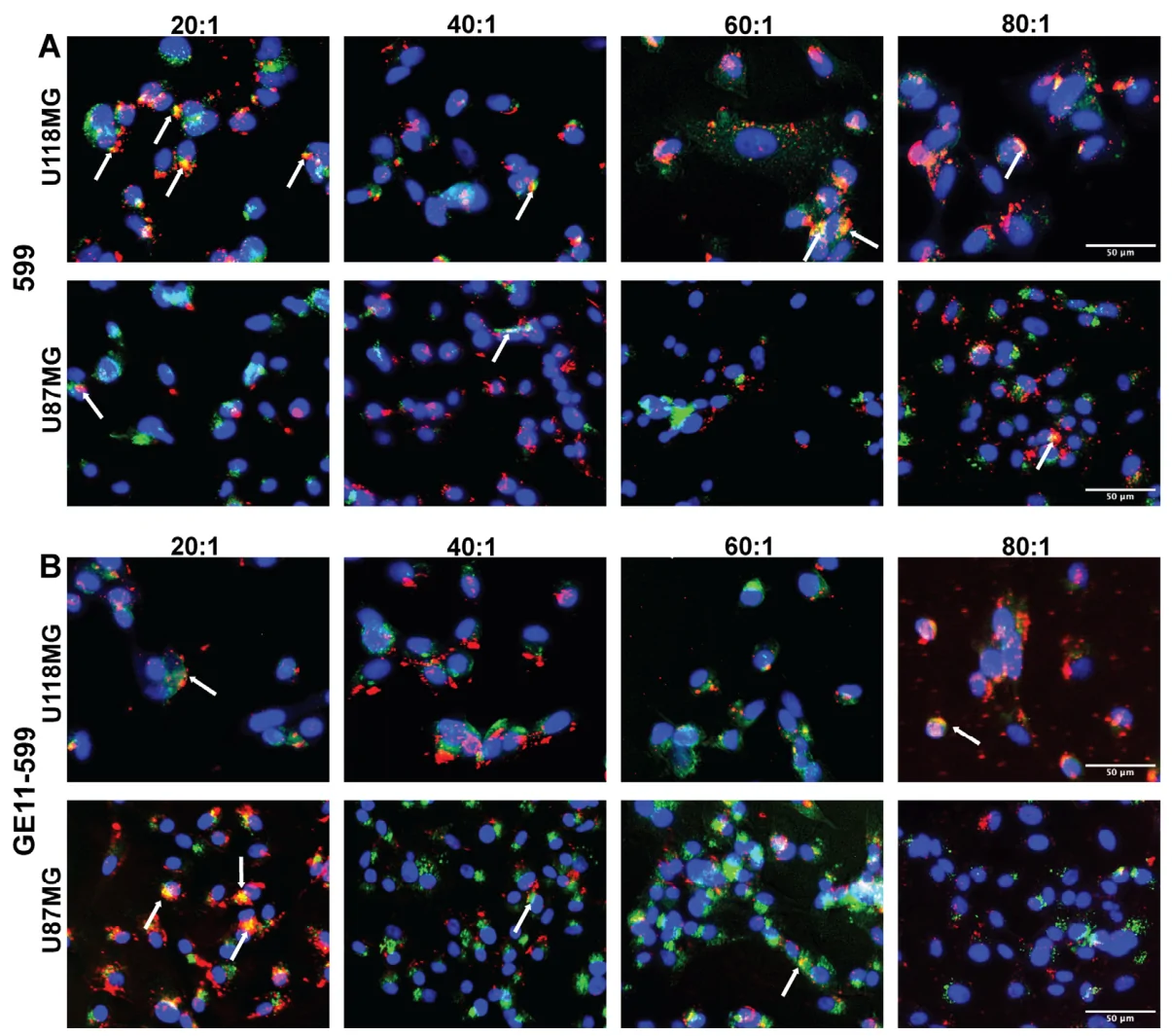
**599 and GE11-599 Peptide-Facilitated Endosomal Escape of siRNA in GBM cells.** Immunofluorescent microscopy of early endosome antigen-1 (green) in U118MG and U87MG GBM cells after a 4-h incubation with (**A**) 599 or (**B**) GE11-599 complexed with Cy5-siNT#5 (red). Cell nuclei were stained with Hoescht-33342 (blue). Arrows indicate instances of colocalization. Scale bar = 50 μm.

An essential aspect of effective siRNA delivery is endosomal escape into the cytoplasm, which enables bioactivity. To assess endosomal escape facilitated by the peptides, immunofluorescent imaging was used to investigate colocalization between internalized siRNA and the endosome, comparing the localization of GE11R9, 599, and GE11-599 peptide complexes. Following treatment with peptide–Cy5-siNT#5, we evaluated instances of endosomal colocalization, with white arrows indicating regions where siRNA remained sequestered within the endosome. In contrast, spatially distinct green and red pixels indicate successful endosomal escape of the cargo. Minimal colocalization was observed for 599 and GE11-599 in both U118MG and U87MG cells, particularly at higher N:P ratios, indicating that these peptides effectively facilitate endosomal escape of siRNA (Figure 6A,B). To further characterize endosomal escape across formulations and N:P ratios, threshold Manders’s colocalization coefficients between EEA1 and labeled siRNA were quantified in U118MG (Figure S8A) and U87MG (Figure S8B) cells. No statistically significant differences in colocalization were observed between 599 and GE11-599 at any N:P ratio in either cell line, nor were significant differences observed across N:P ratios within each peptide. These results indicate that the degree of siRNA colocalization with early endosomes is largely consistent across peptide formulations and N:P ratios under the tested conditions. In contrast, GE11R9 exhibited lower overall siRNA signal intensity, rendering it unquantifiable. However, immunofluorescent microscopy revealed numerous instances of siRNA–endosome colocalization across all N:P ratios after treatment with GE11R9 complexes in both cell lines (Figure S9).

### 3.6. The 599 and GE11-599 Peptides Mediate STAT3 Gene Silencing in Glioblastoma Cell Lines

**Figure 7:**
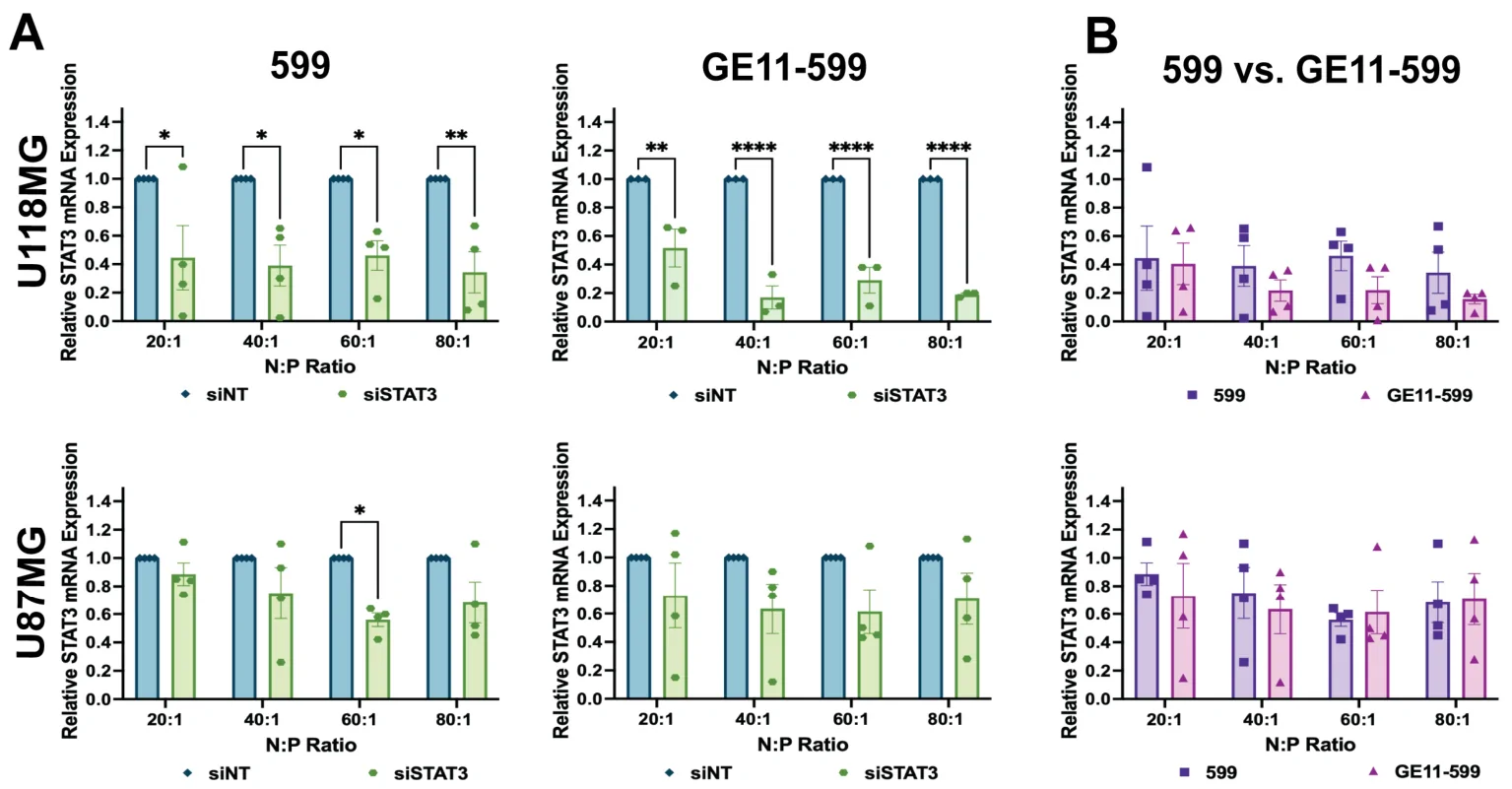
**599- and GE11-599-Mediated *STAT3* Gene Silencing.** (**A**) qPCR analysis of *STAT3* mRNA expression following 48-h treatment with 599 or GE11-599 complexed with siNT or siSTAT3 at varying N:P ratios in U118MG and U87MG cell lines. (**B**) Comparison of *STAT3* mRNA expression after treatments with 599 vs. GE11-599 complexes in U118MG and U87MG cell lines. Data are mean ± SEM of three independent experiments performed in triplicate and analyzed via a two-way ANOVA with Tukey’s multiple comparisons test, where * *p* ≤ 0.05, ** *p* ≤ 0.01, and **** *p* ≤ 0.0001.

To analyze siRNA bioactivity, we examined target gene silencing mediated by peptides complexed with STAT3 siRNA. The GE11R9 peptide showed no significant reductions in gene silencing at any N:P ratio in U118MG and U87MG cell lines (Figure S10). In both U87MG and U118MG cell lines, the 599 peptide complexes reduced *STAT3* mRNA expression at various N:P ratios, indicating efficient siRNA bioactivity. In the U118MG cell line, all ratios of 599 complexes exhibited similar silencing capabilities, with the highest reductions in *STAT3* expression of 61% and 66% at the 40:1 and 80:1 N:P ratios, respectively (Figure 7A). In the U87MG cell line, the 599 peptide complexes exhibited maximal bioactivity at an N:P ratio of 60:1, resulting in a 44% reduction in *STAT3* mRNA expression. The GE11-599 peptide complexes exhibited siRNA bioactivity comparable to the 599 peptide, as evidenced by reduced *STAT3* mRNA expression. In the U118MG cells, the GE11-599 peptide complexes exhibited the highest siRNA bioactivity at the 40:1 and 80:1 N:P ratios, with reductions in *STAT3* expression of 83% and 80%, respectively. In the U87MG cells, the 60:1 GE11-599 ratio was the most effective, resulting in a 39% reduction in *STAT3* expression. When comparing the silencing capabilities of the 599 and GE11-599 peptides, GE11-599 exhibited similar silencing efficiencies across all ratios in both cell lines (Figure 7B). These results indicate that the incorporation of the GE11 targeting region does not hinder the functionality of the 599 fusogenic sequence within the GE11-599 peptide, allowing for necessary endosomal escape and gene silencing.

### 3.7. GE11-599 Peptide-Mediated Silencing of STAT3 Inhibits Cellular Migration

**Figure 8:**
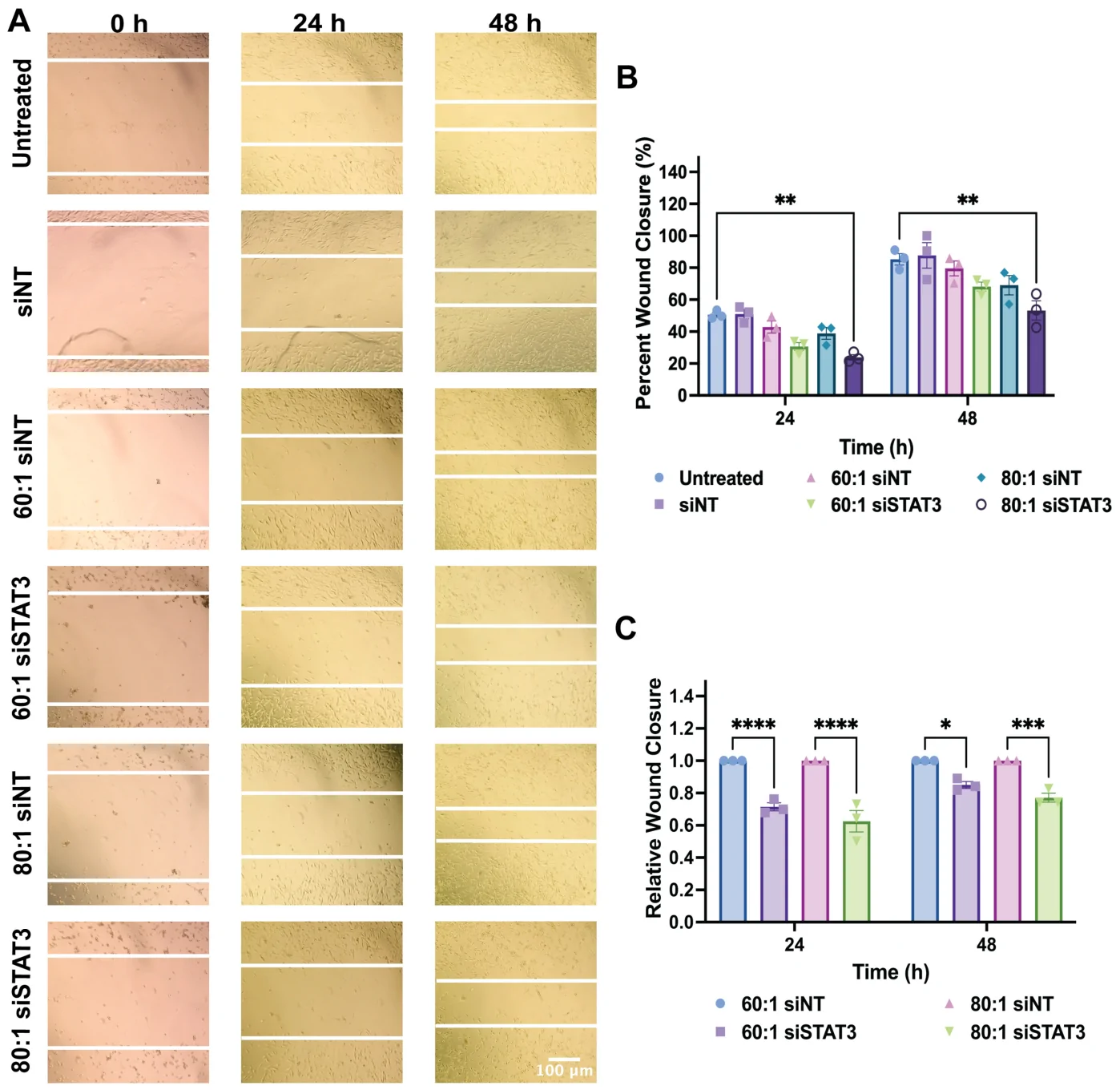
**Scratch Wound-Healing Assay Following GE11-599-mediated *STAT3* Knockdown in U118MG cells.** U118MG cells were treated with GE11-599 peptide complexed with siNT#5 or siSTAT3 at 60:1 and 80:1 N:P ratios for 48 h, after which a scratch was introduced into the cell monolayer and wound was monitored for an additional 48 h. (**A**) Representative phase microscopy imaging at 0-, 24-, and 48-h post-scratch. Scale bar: 100 µm (**B**) Quantification of wound closure using ImageJ and represented as percent wound healing from the initial scratch. (**C**) Relative wound closure was normalized to siNT for each ratio. Data are mean ± SEM of three independent experiments and analyzed via two-way ANOVA with Tukey’s multiple comparisons, where * *p* ≤ 0.05, ** *p* ≤ 0.01, *** *p* ≤ 0.001, and **** *p* ≤ 0.0001.

**Figure 9:**
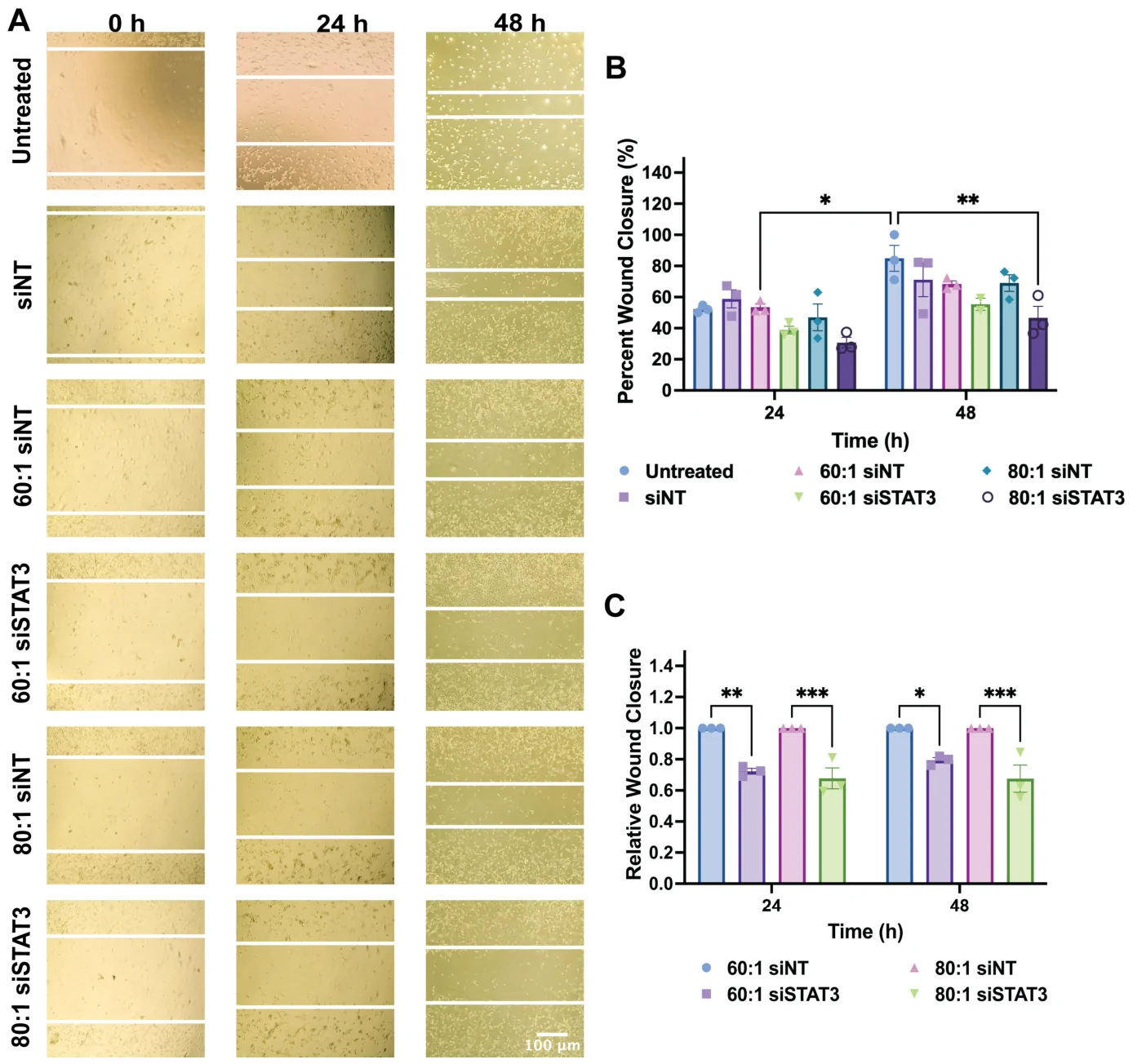
**Scratch Wound-Healing Assay Following GE11-599-mediated *STAT3* Knockdown in U87MG cells.** U118MG cells were treated with GE11-599 peptide complexed with siNT#5 or siSTAT3 at 60:1 and 80:1 N:P ratios for 48 h, after which a scratch was introduced into the cell monolayer and wound was monitored for an additional 48 h. (**A**) Representative phase microscopy imaging at 0-, 24-, and 48-h post-scratch. Scale bar: 100 µm (**B**) Quantification of wound closure using ImageJ and represented as percent wound healing from the initial scratch. (**C**) Relative wound closure was normalized to siNT for each ratio. Data are mean ± SEM of three independent experiments and analyzed via two-way ANOVA with Tukey’s multiple comparisons, where * *p* ≤ 0.05, ** *p* ≤ 0.01, and *** *p* ≤ 0.001.

Because *STAT3* plays a role in cellular migration [27], we conducted a scratch wound-healing assay to examine the effect of silencing *STAT3* on the migration of GBM cells. We treated U118MG and U87MG cells with GE11-599-siNT or GE11-599-siSTAT3 at a 60:1 or 80:1 N:P ratio, as these ratios provided the most significant gene silencing. Following a 48 h treatment, a horizontal scratch was made in the monolayer of the cells (0 h time point), and images were acquired 24- and 48-h post-scratch. Representative images are seen in Figure 8A and Figure 9A for U118MG and U87MG cells, respectively. ImageJ was used to quantify the percentage of wound closure for each time point (Figure 8B and Figure 9B). At 24 h, untreated U118MG cells exhibited 51% wound closure, whereas cells treated with GE11-599-siSTAT3 complexes showed reduced migration, with wound closure of 31% and 24% at 60:1 and 80:1 N:P ratios, respectively. By 48 h, wound closure in untreated cells increased to 85%, while GE11-599-siSTAT3-treated cells reached 68% and 54% closure at a 60:1 and 80:1 ratio, respectively. A similar migration trend was observed in U87MG cells, with approximately 46% wound closure of untreated cells at 24 h, and 39% and 31% closure with GE11-599-siSTAT3 treatment at 60:1 and 80:1 ratios, respectively. After 48 h post-scratch, the untreated U87MG cells exhibited 85% wound closure, compared to 55% and 47% closure at 60:1 and 80:1 ratios, respectively. When normalizing to siNT at each N:P ratio, *STAT3* silencing mediated by the GE11-599 complex reduced cell migration at 48 h in both GBM cell lines. In U118MG cells, migration was reduced by approximately 15% and 23% at the 60:1 and 80:1N:P ratios, respectively (Figure 8C). Similarly, in the U87MG cells, GE11-599-siSTAT3 reduced migration by approximately 20% at the 60:1 ratio and 32% at the 80:1 ratio at the 48 h time point (Figure 9C). These results confirm that *STAT3* silencing confers an inhibitory effect on GBM cell migration.

**Figure 10:**
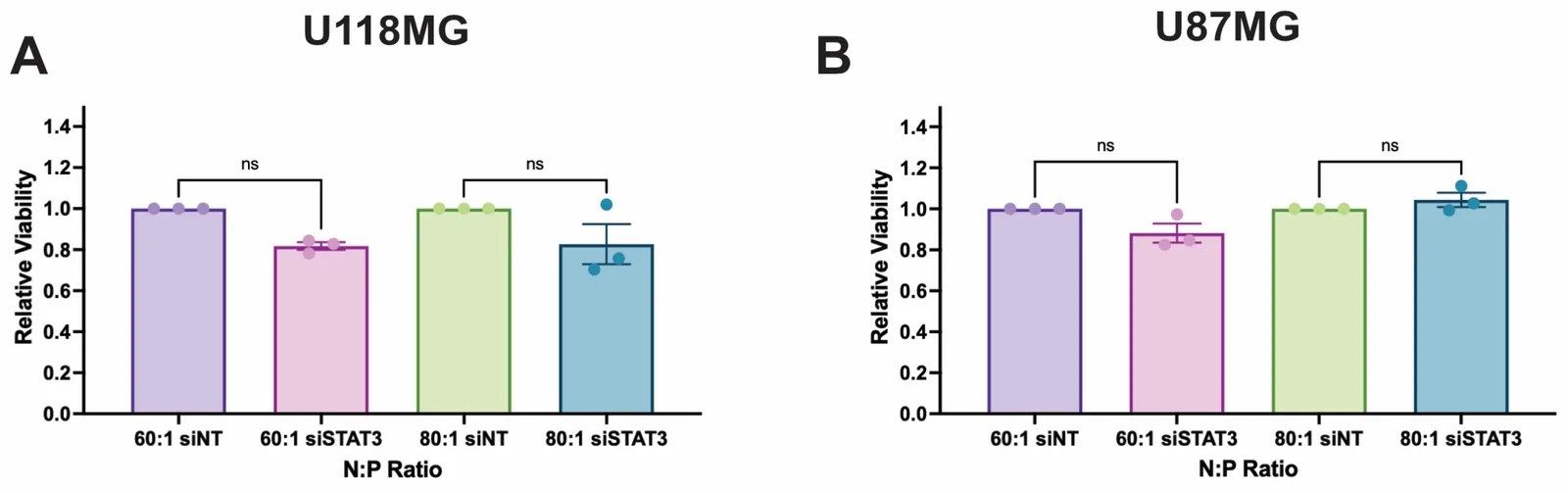
**Viability Assay Following GE11-599-mediated STAT3 Knockdown in GBM cells.** Cellular viability after 48-h treatment with GE11-599-siNT#5 or -siSTAT3 complexes at 60:1 and 80:1 N:P ratios in (**A**) U118MG and (**B**) U87MG cells. Data are mean ± SEM of three independent experiments performed in triplicate and analyzed via two-way ANOVA using Tukey’s multiple comparisons test, where ns = not significant compared to siNT at each N:P ratio.

To confirm that the observed reduction in migration was not attributable to cytotoxic effects, cell viability was assessed in parallel to examine the effects of STAT3 silencing on GBM cell viability. We treated U118MG and U87MG cells with GE11-599-siNT or GE11-599-siSTAT3 at N:P ratios of 60:1 or 80:1 for a 48-h treatment. In U118MG cells (Figure 10A) and U87MG (Figure 10B) cells, GE11-599-mediated STAT3 silencing showed no statistical differences in cellular viability relative to siNT at each ratio. These results indicate that the reduced wound closure reflects migration inhibition rather than cellular cytotoxicity effects.

## 4. Discussion

RNAi has therapeutic potential to treat many diseases through its specific gene targeting; however, effective delivery remains a primary barrier limiting its clinical translation. There are currently six FDA-approved siRNA-based therapies, all of which target disease of the liver [28]. The majority of therapies conjugate siRNA to a sugar molecule, N-acetylgalactosamine (GalNAc), which targets the asialoglycoprotein receptor on liver cells for delivery [29]. While this method has proved effective, expanding siRNA therapies beyond hepatic applications poses challenges and requires the development of new delivery systems. Non-viral delivery systems used for siRNA include lipid nanoparticles, polymeric nanoparticles, and metallic nanoparticles; thus, recent research has focused on employing peptides conjugated with these systems [30,31,32,33]. This combination often improves delivery efficiency through increased membrane penetration, endosomal escape, and target specificity [34,35,36]. Peptide-based delivery systems for siRNA are typically unilateral in function, overcoming a single delivery barrier. Thus, advanced peptide designs are necessary to function effectively as standalone delivery systems. Current research investigating combination sequences typically involves combining targeting sequences with cell-penetrating sequences [37,38]. While these combinations effectively address certain challenges, endosomal entrapment remains a significant hurdle for siRNA delivery. To overcome this hurdle, this study investigates a multifunctional tandem peptide design that combines targeting and fusogenic sequences to facilitate cell-specific internalization and enhanced endosomal escape of RNAi cargo, an area that has been largely unexplored to date.

We investigated the GE11R9-targeting sequence, the 599 fusogenic sequence, and our multifunctional tandem peptide sequence, GE11-599, for siRNA delivery to GBM cells. Each sequence was designed to incorporate a nona(D-arginine) tail for electrostatic complexation with siRNA. Characterization of peptide–siRNA complexes confirmed that each peptide complexes with siRNA and protects siRNA from degradation in physiological and harsher degradative conditions. Additionally, peptide–siRNA complexes were positively charged and formed monodisperse nanoparticles with diameters of less than 200 nm. These nanoparticle characteristics and protective capabilities make them ideal delivery systems for improving the circulation time of siRNA, as nanoparticle sizes between 100–200 nm generally avoid renal clearance and reticuloendothelial system uptake [39]. GE11R9 required a higher N:P ratio for complete complexation and had lower protection capabilities in both degrative environments compared to the 599 and GE11-599 peptide, likely due to the lower surface charge leading to less shielding against degradation [40,41]. Despite the longer amino acid sequence of the GE11-599 peptide, its NP size when complexed with siRNA was smaller or similar in size to the other peptides with shorter sequences. We hypothesize that the size of GE11-599-siRNA NPs is due to the increased charge interactions, allowing for more compact particle formation [42]. Additionally, in vitro studies demonstrated minimal cytotoxicity in U118MG and U87MG GBM cells, as well as in HEK-293 and HMC3 healthy cell lines, at N:P ratios up to 80:1 for all peptide formulations, supporting the overall cytocompatibility of the delivery system.

Following the characterization of peptide/siRNA complexes, we examined the ability of the GE11-599 peptide to mediate efficient delivery of siRNA into GBM cells. Cellular internalization studies indicated successful internalization of all three peptide formulations in U118MG and U87MG GBM cells. Notably, when examining fluorescent microscopy images, we observed differing intracellular localizations of siRNA depending on the peptide. When delivered with the 599 peptide, siRNA localized within the perinuclear cytoplasmic region. However, when delivered with the GE11R9 and GE11-599 peptides, siRNA appeared to localize within the nuclei, as evidenced by the colocalization of these fluorescent signals. We hypothesize that by targeting EGFR with the GE11R9 and GE11-599 peptides, we are utilizing the native intracellular trafficking of EGFR for nuclear translocation [43,44]. A previous study investigated internalization of EGFR-targeted Fe_3_O_4_@TiO_2_ nanoparticles and their nuclear translocation in EGFR-expressing HeLa cells [45]. Yuan et al. determined that conjugating their nanoparticle with a peptide nanoconjugate, which performed most similarly to the native EGF ligand, could maintain interactions between EGFR and the karyopherin protein family, facilitating nuclear translocation [45]. Further studies should determine whether the GE11R9 peptide follows the same pathway. Of note, the internalization of siRNA into both GBM cell lines was significantly higher for the 599 and GE11-599 peptides compared to the GE11R9 peptide at a 20:1 N:P ratio, likely due to the partial complexation of GE11R9 with siRNA at this ratio, resulting in inadequate protection of siRNA from degradation. In U118MG cells, all three peptides facilitated comparable levels of internalization at the 40:1–80:1 N:P ratios. However, in the U87MG cells, internalization facilitated by the GE11R9 peptide was more ratio-dependent, which may be due to lower EGFR expression in U87MG compared to U118MG cells [46,47]. By adding the fusogenic region, the GE11-599 peptide enhanced siRNA internalization compared to the GE11R9 peptide alone due to its increased hydrophobicity, which facilitates cellular interaction and internalization [48]. These results were confirmed when evaluating the MFI for internalization. Across all ratios, GE11R9 consistently produced low MFI values, whereas 599 and GE11-599 displayed comparable MFI, indicating that incorporation of the GE11R9-targeting sequence with the 599 peptide does not diminish siRNA internalization and supports a combined functional effect of the two sequence domains. Notably, for both 599 and GE11-599, MFI was the highest at the 20:1 N:P ratio and declined at higher ratios, despite comparable uptake efficiency across all ratios. Because signal quenching was not observed under these conditions, this trend is unlikely to reflect an artifact of fluorophore packing density. Instead, we hypothesize that excess uncomplexed peptide at higher N:P ratios may compete with labeled complexes for cell-surface binding and entry pathways, thereby limiting the quantity of complex internalized per cell without necessarily altering the number of cells that take up material. Because this trend was observed for both 599 and GE11-599, it likely reflects a physicochemical, peptide-driven effect rather than a receptor-specific effect.

Targeted drug delivery is essential to ensure the cargo reaches the desired cells with minimal off-target accumulation. EGFR is a transmembrane protein overexpressed in many cancers, including GBM, lung, pancreatic, and ovarian cancers [49]. Therefore, EGFR-targeting moieties have been conjugated to nanoparticle delivery systems to enhance accumulation in EGFR-overexpressing cancers [50,51]. EGFR is expressed in 50–60% of GBM tissues and is minimally expressed in healthy brain tissue, making it an ideal target for our delivery system [52,53]. Therefore, we utilized the GE11 sequence, which was identified through phage display, to target EGFR without activating the receptor to induce a mitogenic effect [15]. GE11 has been conjugated to nanoparticles for targeted delivery of gene therapy and image probes, which are used for applications within GBM and radiolabeling [54]. Using receptor-blocking experiments, we confirmed that GE11R9 and GE11-599 use EGFR-receptor-mediated endocytosis as an internalization route in U87MG and U118MG GBM cell lines. Furthermore, we observed that siRNA internalization by the 599 peptide was not affected when EGFR was blocked, confirming its receptor-independent internalization [20,55]. GE11 has also been shown to penetrate the blood–brain barrier, suggesting that incorporating this peptide in our tandem design could yield similar results, enhancing the potential for clinical translation of the tandem peptide for RNA delivery to treat GBM [56].

A significant barrier to nanoparticle delivery is the lack of endosomal escape, which results in ineffective cargo release into the cytoplasm due to entrapment [13,57,58]. Thus, we investigated the capability of the GE11-599 peptide to facilitate endosomal escape. The 599 sequence was designed with a fusogenic region derived from INF-7 and a short cell-penetrating sequence for complexation with siRNA. Previous work has demonstrated that the 599 peptide can induce significant endosomal escape and oncogene silencing in both in vitro and in vivo models of oral cancer [19,20,59]. Therefore, we investigated its endosomal escape and gene silencing capability alone and within the tandem sequence in GBM cells. Immunofluorescent imaging revealed minimal colocalization between siRNA and endosomes, indicating that 599 and GE11-599 successfully escaped the endosome and had similar threshold Mander’s coefficients for endosomal escape semi-quantitative analysis. This endosomal escape results in the delivery of bioactive siRNAs, resulting in up to 76% and 40% silencing of *STAT3* mRNA in U118MG and U87MG cells, respectively. Although the 20:1 ratio of 599 and GE11-599 showed high cargo delivery intensity in internalization studies, it resulted in similar or reduced gene silencing, particularly in U87MG cells. This finding suggests that cellular uptake alone does not dictate functional siRNA delivery and that additional intracellular barriers likely influence silencing efficacy. Reduced gene silencing may suggest greater endosomal entrapment at these ratios, further highlighting endosomal escape as a rate-limiting step in effective siRNA delivery [58,60,61]. Increasing the N:P ratio increases the relative abundance of fusogenic peptide, which may enhance membrane destabilization and endosomal escape capacity. However, because EEA1 labels early endosomes, these measurements do not capture subsequent trafficking events that ultimately determine productive cytosolic delivery. Thus, the enhanced gene silencing observed at the 80:1 N:P ratio may result from more efficient progression through downstream intracellular processes. Future studies employing additional endosomal maturation markers, such as LAMP-1, along with temporal trafficking analyses, will be valuable for delineating the intracellular fate of these nanocomplexes and identifying the mechanisms responsible for improved silencing at higher N:P ratios. Nonetheless, these results demonstrate that the GE11-599 peptide retains the functionality of the 599 sequence, as evidenced by its ability to facilitate endosomal escape and deliver bioactive siRNA for gene silencing.

*STAT3* plays a role in various cellular functions associated with cancer progression, including proliferation, invasion, and cellular migration [62]. It regulates matrix metalloproteinases, including MMP2 and MMP9, to promote cellular migration, thereby aiding GBM survival [63]. *STAT3* has been investigated as a therapeutic target for GBM due to its widespread role and upregulation in patients [22,64,65]. Esposito et al. utilized the Gin4.T aptamer to facilitate siSTAT3 delivery, successfully decreasing migration by 30% in U87MG cells after a 24-h treatment [66]. Therefore, we investigated the migration capacity of GBM cells through a scratch wound-healing assay after silencing *STAT3* with the GE11-599 peptide complexes. We chose the 60:1 and 80:1 N:P ratios for downstream analysis, as these were the top-performing ratios in gene-silencing studies. Following treatment with GE11-599-siSTAT3 complexes, we observed a reduction of up to 40% in the migration of U118MG and U87MG cells compared to cells treated with GE11-599-siNT 24- and 48-h post-scratch. Notably, cell viability remained unaffected under these same treatment conditions, confirming that the migratory inhibition was a specific consequence of STAT3 silencing rather than a secondary effect of reduced cell survival. Overall, these results demonstrate the therapeutic potential of GE11-599-mediated oncogene silencing in reducing GBM cancer progression.

## 5. Conclusions

In conclusion, we developed a novel multifunctional peptide capable of targeting glioblastoma cells for efficient internalization and facilitating endosomal escape of its cargo. We leveraged GE11, an EGFR-targeting sequence, and 599, a fusogenic sequence, to design a nanocarrier capable of delivering bioactive siRNA. Our GE11-599 tandem peptide formed positively charged, monodisperse nanocomplexes with siRNA and protected siRNA in physiological and harsher degradative conditions. Additionally, the tandem peptide exhibited minimal cytotoxicity in GBM and healthy cell lines. The GE11-599 peptide successfully internalized siRNA into GBM cells through cell-specific targeting of EGFR. Furthermore, the GE11-599 peptide facilitated the escape of siRNA from the endosome, allowing for efficient silencing of the target oncogene, *STAT3*. Additionally, we demonstrated the therapeutic efficacy of our system by confirming that *STAT3* silencing reduced migration in two GBM cell lines. Our study demonstrates the successful design of a multifunctional peptide that integrates two distinct functional sequences, enabling the delivery system to overcome multiple barriers associated with siRNA bioactivity.

## Data Availability

The original contributions presented in this study are included in the article/Supplementary Materials. Further inquiries can be directed to the corresponding author.

## Supplementary Materials

The following supporting information can be downloaded at: https://www.mdpi.com/article/10.3390/pharmaceutics18091086/s1, Figure S1: GE11R9 Characterization. (A) GE11R9 peptide design and electrostatic complexation with siRNA. (B) Gel electrophoresis of GE11R9 at increasing N:P ratios with intact, free siRNA indicated by white banding. (C) Protection assays of GE11R9 peptide–siRNA complexes in RNase-free water (−) and 50% FBS or 0.15 mg/mL of RNase A (+); Figure S2: 599 Characterization. (A) 599 peptide design and electrostatic complexation with siRNA. (B) Gel electrophoresis of 599 at increasing N:P ratios with intact, free siRNA indicated by white banding. (C) Protection assays of 599 peptide–siRNA complexes in RNase-free water (−) and 50% FBS or 0.15 mg/mL of RNase A (+); Figure S3: Dynamic light-scattering size distribution profiles of GE11R9, 599, and GE11-599 complexes at the 80:1 N:P ratio. Representative size distribution plots by intensity for GE11R9 (top), 599 (middle), and GE11-599 (bottom) complexes formed at an 80:1 N:P ratio in H_2_O. Each plot displays three independent measurements overlaid by the particle-size distribution for each formulation; Figure S4: GE11-599 Cytocompatibility in Healthy Cell Lines. Cellular viability after 48-h treatment with GE11-599-siNT#5 complexes at varying N:P ratios in (A) HEK293-GFP and (B) HMC3 cells. Data are mean ± SEM of three independent experiments performed in triplicate, where ns = not significant compared to untreated cells analyzed via one-way ANOVA using Dunnett’s multiple comparisons test; Figure S5: GBM Cell Flow Cytometry Gating. Representative figure of the flow cytometry gating strategy for (A) untreated cells and (B) cells positive for siNT-Cy5. U118MG and U87MG cells are first-gated (Live Cell Population), and the region of cells positive for siNT-Cy5 (R1) is identified by eliminating negative cells using the untreated cell population; Figure S6: Fluorophore quenching control for Cy5-labeled siRNA across N:P ratios. Dy547-labeled siRNA was complexed with GE11-599 at N:P ratios of 20:1, 40:1, 60:1, and 80:1, and fluorescence intensity was measured using a BioTek Synergy Gen5 plate reader using the red fluorescence filter cube. Data represent the mean ± SEM of three independent experiments, analyzed by one-way ANOVA with Tukey’s multiple comparisons test, where ns = not significant; Figure S7: EGFR Competitive Binding of Peptide–siRNA Complexes. ImageJ quantification of peptide–Cy5-siNT#5 fluorescence intensity in (A) U118MG and (B) U87MG cells, where cells were pretreated with either PBS (−EGFR antibody) or a Cetuximab biosimilar (+EGFR antibody) for 1 h, followed by treatment with peptide–Cy5-siNT#5 complexes for 3 h and imaged via fluorescent microscopy. Data are mean ± SEM of three independent experiments and analyzed via a two-way ANOVA with Tukey’s multiple comparisons test, where ** *p* ≤ 0.01; Figure S8: Threshold Manders’s colocalization coefficients between EEA1 and 599- and GE11-599-Cy5 siRNA, in GBM cells. Colocalization between early endosomes (EEA1) and Cy5-labeled siRNA delivered by 599 or GE11-599 complexes was quantified using threshold Manders’s coefficients in (A) U118MG and (B) U87MG cells across N:P ratios. Data are mean ± SEM of three independent experiments and analyzed via two-way ANOVA with Tukey’s multiple comparisons test; Figure S9: GE11R9 Peptide-Facilitated Endosomal Escape of siRNA in GBM cells. Immunofluorescent microscopy of early endosome antigen-1 (green) in U118MG and U87MG GBM cells after a 4-h incubation with GE11R9 complexed with Cy5-siNT#5 (red). Cell nuclei were stained with Hoescht-33342 (blue). Arrows indicate instances of colocalization (yellow). Scale bar = 50 µm; Figure S10: GE11R9-Mediated *STAT3* Gene Silencing. qPCR analysis of *STAT3* mRNA expression following 48-h treatment with GE11-R9 complexed with siNT or siSTAT3 at varying N:P ratios in U118MG and U87MG cell lines. Data are mean ± SEM of three independent experiments performed in triplicate and analyzed via a two-way ANOVA with Tukey’s multiple comparisons test.

## Abbreviations

The following abbreviations are used in this manuscript:

GBM	Glioblastoma
RNAi	RNA interference
siRNA	Short interfering RNA
mRNA	Messenger RNA
STAT3	Signal Transducer and Activator of Transcription 3
EGFR	Epidermal Growth Factor Receptor
PDI	Polydispersity Index
SEM	Standard Error of the Mean
N:P	Amine:phosphate ratio
EEA1	Early endosome antigen 1
SDS	Sodium dodecyl sulfate
MFI	Mean Fluorescence Intensity
