# Supplementary material for: An EGFR-Targeted Fusogenic Tandem Peptide for siRNA Delivery in Glioblastoma
Source: Pharmaceutics. 2026 Aug 28;18(9):1086. doi: 10.3390/pharmaceutics18091086 (PMC13610838; doi:10.3390/pharmaceutics18091086)
Supplement: Supplementary file 1 [file pharmaceutics-18-01086-s001.zip › pharmaceutics-4190711-supplementary.pdf]

Article

An EGFR-Targeted Fusogenic Tandem Peptide for siRNA Delivery in Glioblastoma

Jessica R. Boulos, Karen E. Russi, Jordan Kinnitt, Daphne Gomez Escudero, Tyler Willis, Jorrian Abadeer, Emalee Mann, Aaron Cristina Anderson, Angela Alexander-Bryant*

Department of Bioengineering, Nanobiotechnology Laboratory, Clemson University, Clemson, South Carolina 29631, United States

* Correspondence: angelaa@clemson.edu ; Tel.: 864-656-5232

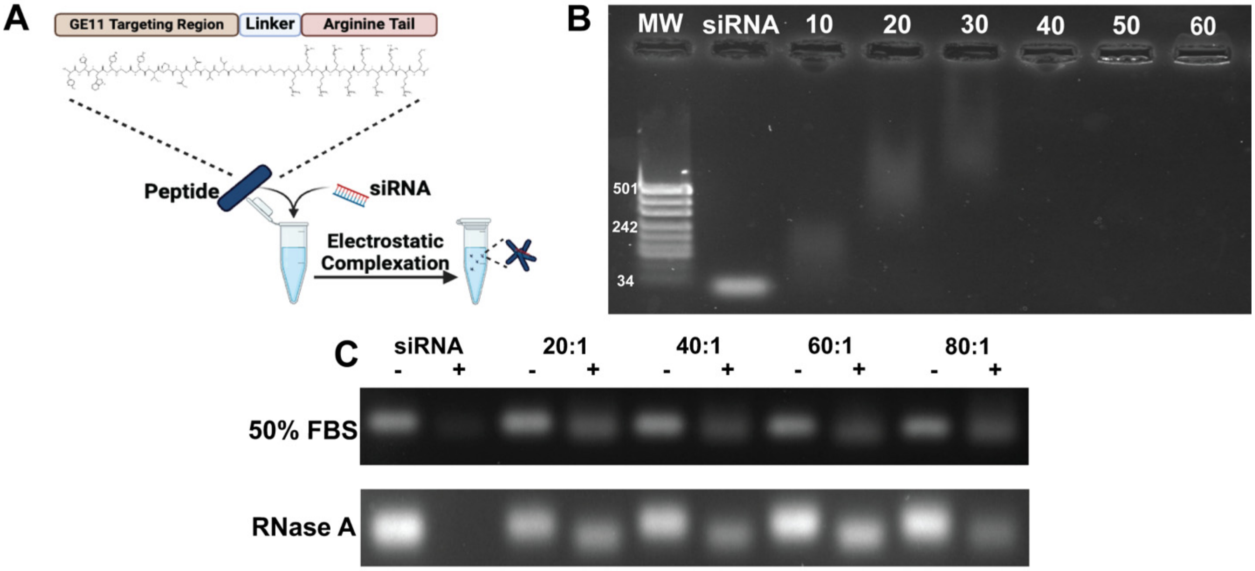

Figure S1. GE11R9 Characterization. (A) GE11R9 peptide design and electrostatic complexation with siRNA. (B) Gel electrophoresis of GE11R9 at increasing N:P ratios with intact, free siRNA indicated by white banding. (C) Protection assays of GE11R9 peptide-siRNA complexes in RNase-free water (-) and 50%FBS or 0.15 mg/mL of RNase A (+).

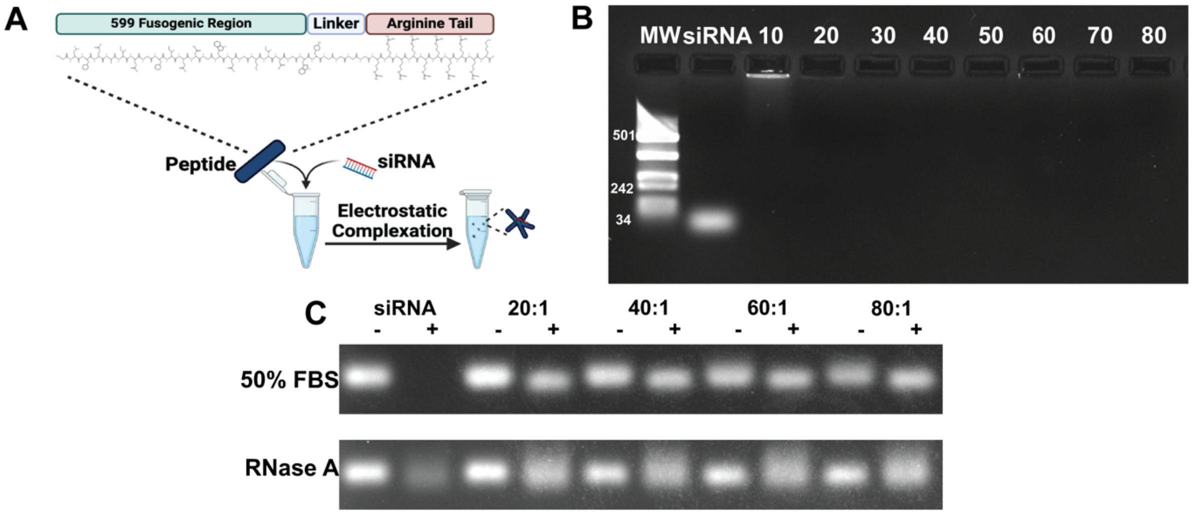

Figure S2. 599 Characterization. (A) 599 peptide design and electrostatic complexation with siRNA. (B) Gel electrophoresis of 599 at increasing N:P ratios with intact, free siRNA indicated by white banding. (C) Protection assays of 599 peptide-siRNA complexes in RNase-free water (-) and 50%FBS or 0.15 mg/mL of RNase A (+).

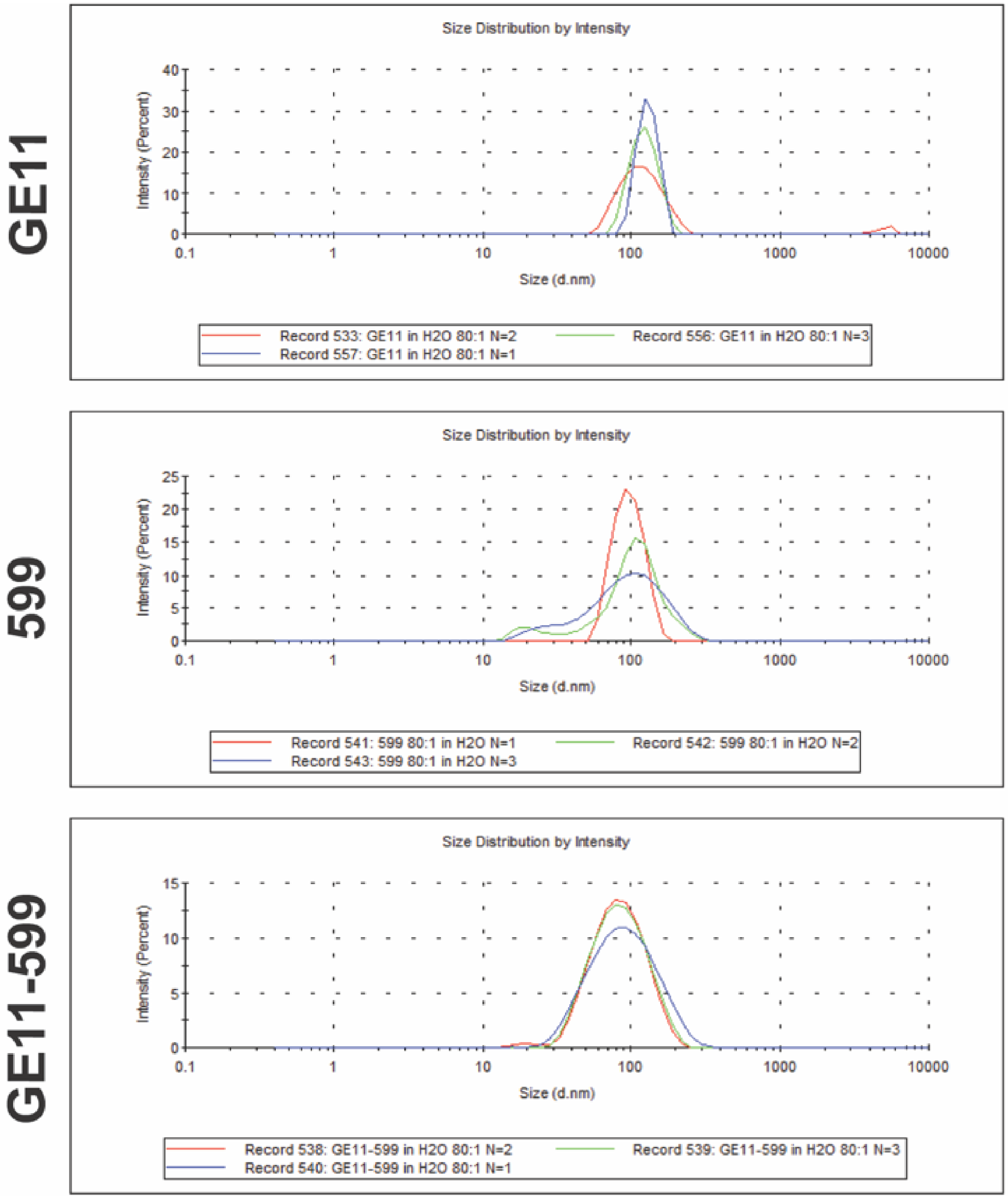

Figure S3. Dynamic light scattering size distribution profiles of GE11R9, 599, and GE11-599 complexes at the 80:1 N:P ratio. Representative size distribution plots by intensity for GE11R9 (top), 599 (middle), and GE11-599 (bottom) complexes formed at an 80:1 N:P ratio in H_2O. Each plot displays three independent measurements overlaid by the particle-size distribution for each formulation.

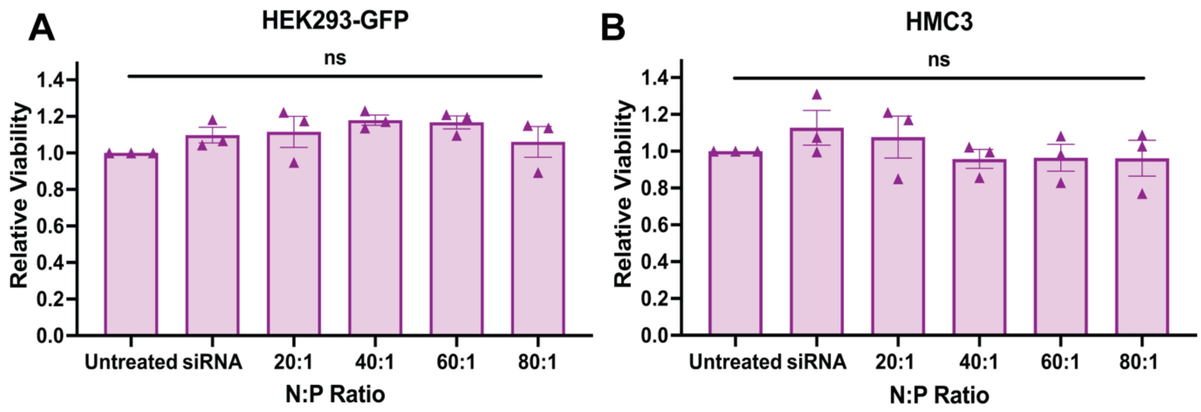

Figure S4. GE11-599 Cytocompatibility in Healthy Cell Lines. Cellular viability after 48-h treatment with GE11-599-siNT#5 complexes at varying N:P ratios in (A) HEK293-GFP and (B) HMC3 cells. Data are mean ± SEM of three independent experiments performed in triplicate, where ns=not significant compared to untreated cells analyzed via one-way ANOVA using Dunnett's multiple comparisons test.

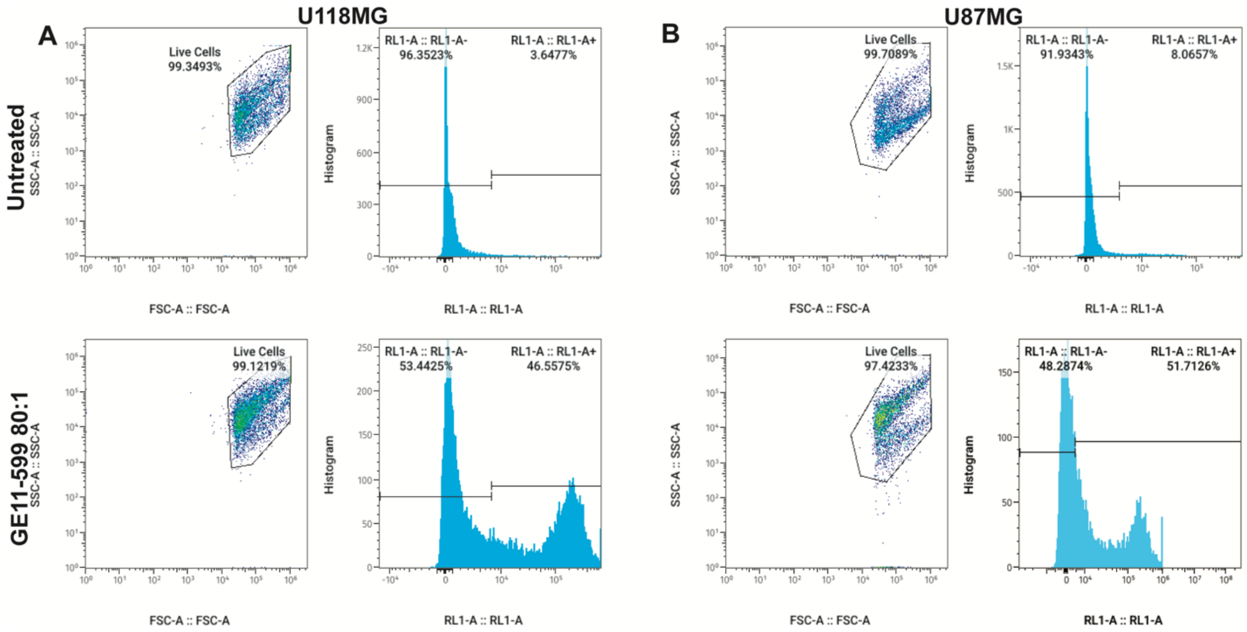

Figure S5. GBM Cell Flow Cytometry Gating. Representative figure of the flow cytometry gating strategy for (A) untreated cells and (B) cells positive for siNT-Cy5. U118MG and U87MG cells are first gated (Live Cell Population), and the region of cells positive for siNT-Cy5 (R1) is identified by eliminating negative cells using the untreated cell population.

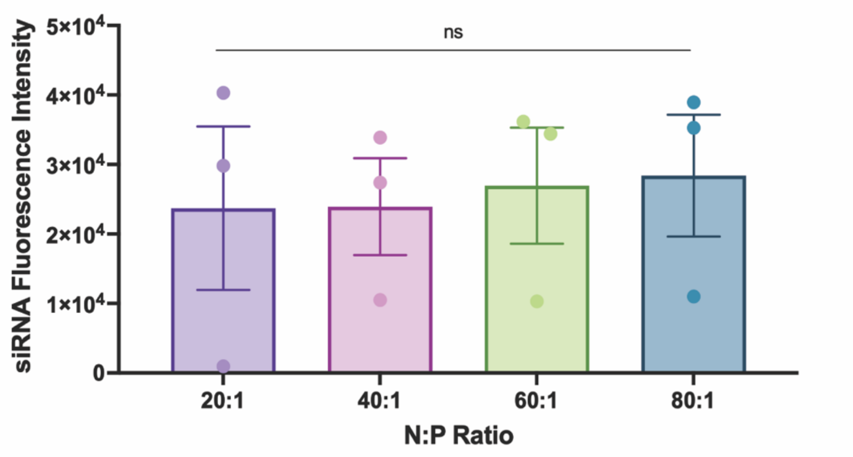

Figure S6. Fluorophore quenching control for Cy5-labeled siRNA across N:P ratios. Dy547-labeled siRNA was complexed with GE11-599 at N:P ratios of 20:1, 40:1, 60:1, and 80:1, and fluorescence intensity was measured using a BioTek Synergy Gen5 plate reader using the red fluorescence filter cube. Data represent the mean ± SEM of three independent experiments, analyzed by one-way ANOVA with Tukey's multiple comparisons test, where ns=not significant.

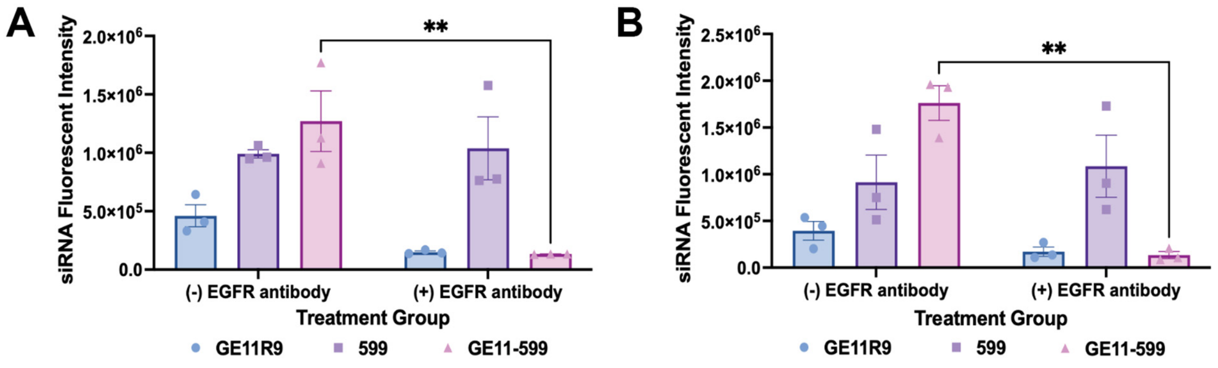

Figure S7. EGFR Competitive Binding of Peptide-siRNA Complexes. ImageJ quantification of peptide-Cy5-siNT#5 fluorescence intensity in (A) U118MG and (B) U87MG cells, where cells were pretreated with either PBS (- EGFR antibody) or a Cetuximab biosimilar (+ EGFR antibody) for 1 h, followed by treatment with peptide-Cy5-siNT#5 complexes for 3 h and imaged via fluorescent microscopy. Data are mean ± SEM of three independent experiments and analyzed via a two-way ANOVA with Tukey's multiple comparisons test, where **$p \leq 0.01$.

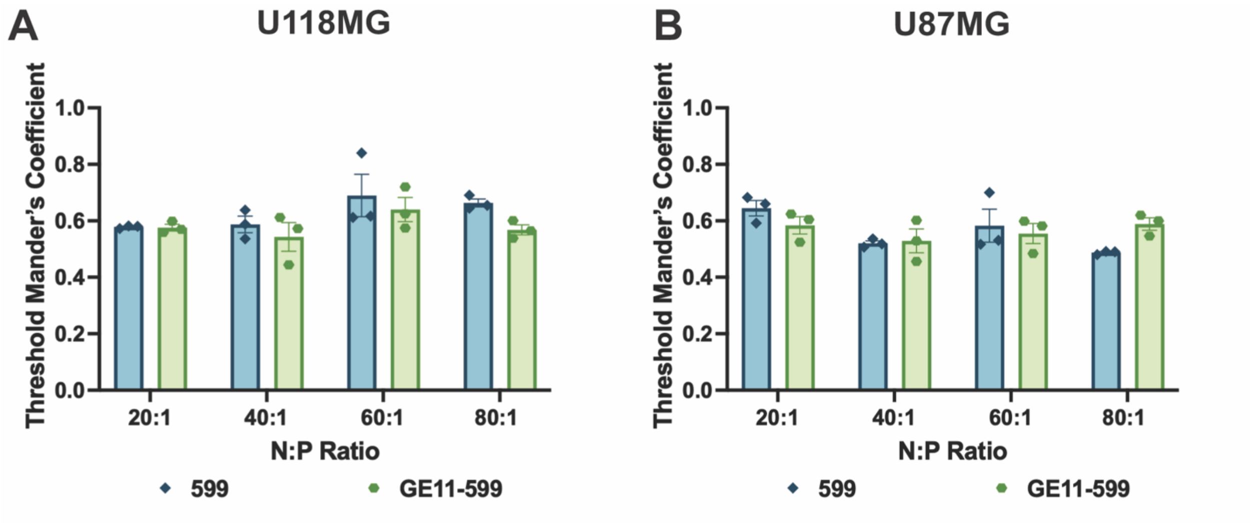

Figure S8. Threshold Manders's colocalization coefficients between EEA1 and 599- and GE11-599-Cy5 siRNA, in GBM cells. Colocalization between early endosomes (EEA1) and Cy5-labeled siRNA delivered by 599 or GE11-599 complexes was quantified using threshold Manders's coefficients in (A) U118MG and (B) U87MG cells across N:P ratios. Data are mean ± SEM of three independent experiments and analyzed via two-way ANOVA with Tukey's multiple comparisons test.

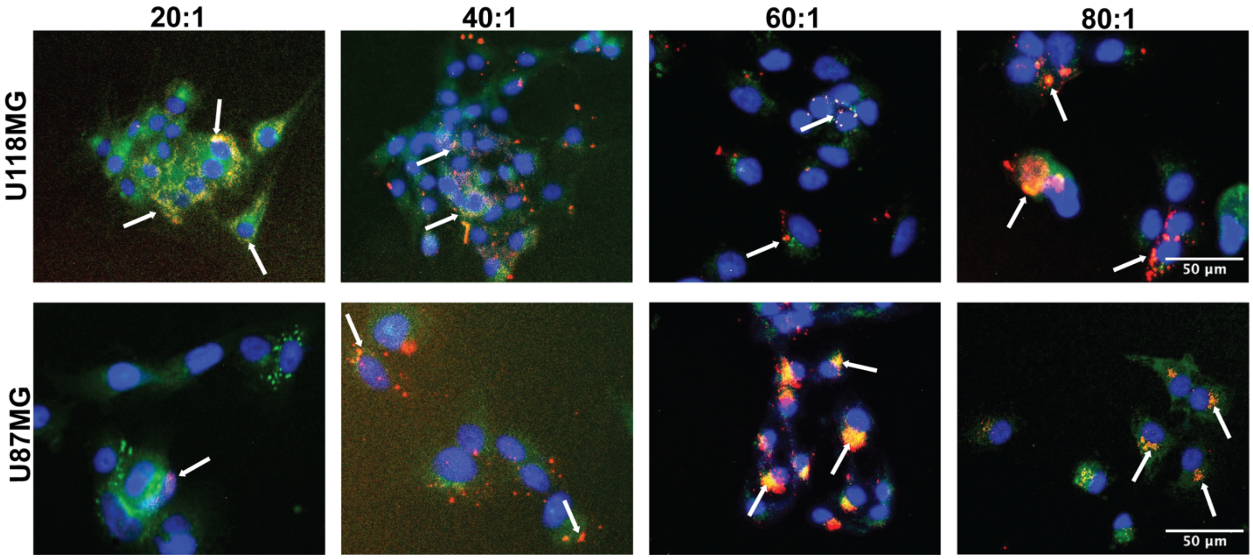

Figure S9. GE11R9 Peptide-Facilitated Endosomal Escape of siRNA in GBM cells. Immunofluorescent microscopy of early endosome antigen-1 (green) in U118MG and U87MG GBM cells after a 4-h incubation with GE11R9 complexed with Cy5-siNT#5 (red). Cell nuclei were stained with Hoescht-33342 (blue). Arrows indicate instances of colocalization (yellow). Scale bar = 50 µm.

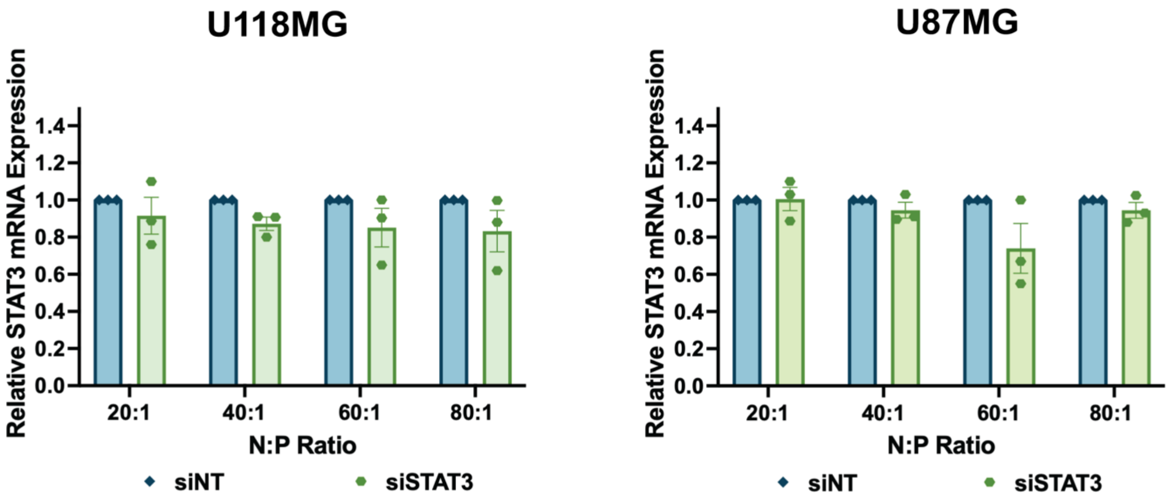

Figure S10. GE11R9-Mediated *STAT3* Gene Silencing. qPCR analysis of *STAT3* mRNA expression following 48-h treatment with GE11-R9 complexed with siNT or siSTAT3 at varying N:P ratios in U118MG and U87MG cell lines. Data are mean ± SEM of three independent experiments performed in triplicate and analyzed via a two-way ANOVA with Tukey's multiple comparisons test.
